# DivIVA Controls Progeny Morphology and Diverse ParA Proteins Regulate Cell Division or Gliding Motility in *Bdellovibrio bacteriovorus*

**DOI:** 10.3389/fmicb.2020.00542

**Published:** 2020-04-21

**Authors:** David S. Milner, Luke J. Ray, Emma B. Saxon, Carey Lambert, Rob Till, Andrew K. Fenton, Renee Elizabeth Sockett

**Affiliations:** ^1^Laboratory C15, Division of Infections, Immunity and Microbes, School of Life Sciences, University of Nottingham, Nottingham, United Kingdom

**Keywords:** predatory bacteria, *Bdellovibrio*, ParAB, DivIVA, septation, cell-morphology

## Abstract

The predatory bacterium *B. bacteriovorus* grows and divides inside the periplasm of Gram-negative bacteria, forming a structure known as a bdelloplast. Cell division of predators inside the dead prey cell is not by binary fission but instead by synchronous division of a single elongated filamentous cell into odd or even numbers of progeny cells. *Bdellovibrio* replication and cell division processes are dependent on the finite level of nutrients available from inside the prey bacterium. The filamentous growth and division process of the predator maximizes the number of progeny produced by the finite nutrients in a way that binary fission could not. To learn more about such an unusual growth profile, we studied the role of DivIVA in the growing *Bdellovibrio* cell. This protein is well known for its link to polar cell growth and spore formation in Gram-positive bacteria, but little is known about its function in a predatory growth context. We show that DivIVA is expressed in the growing *B. bacteriovorus* cell and controls cell morphology during filamentous cell division, but not the number of progeny produced. Bacterial Two Hybrid (BTH) analysis shows DivIVA may interact with proteins that respond to metabolic indicators of amino-acid biosynthesis or changes in redox state. Such changes may be relevant signals to the predator, indicating the consumption of prey nutrients within the sealed bdelloplast environment. ParA, a chromosome segregation protein, also contributes to bacterial septation in many species. The *B. bacteriovorus* genome contains three ParA homologs; we identify a canonical ParAB pair required for predatory cell division and show a BTH interaction between a gene product encoded from the same operon as DivIVA with the canonical ParA. The remaining ParA proteins are both expressed in *Bdellovibrio* but are not required for predator cell division. Instead, one of these ParA proteins coordinates gliding motility, changing the frequency at which the cells reverse direction. Our work will prime further studies into how one bacterium can co-ordinate its cell division with the destruction of another bacterium that it dwells within.

## Introduction

*B. bacteriovorus* is a small predatory bacterium that invades and replicates within other Gram-negative bacteria, forming a rounded structure called a ‘bdelloplast’. Inside the dead prey bacterium, the *Bdellovibrio* cell elongates to form a multiploid filament, before synchronous septation liberates odd or even numbered progeny (Fenton et al., 2010a). Predatory mutants of *B. bacteriovorus* can be saved and cultured slowly by host-independent (HI) axenic growth, requiring an amino acid rich medium (Seidler and Starr, 1969; Cotter and Thomashow, 1992). HI growth also involves septation of a filamentous cell, akin to that in the bdelloplast, although HI cells are pleiomorphic, and division can occur either synchronously or asynchronously (Cotter and Thomashow, 1992; Hobley et al., 2012b). During *Bdellovibrio* predatory growth in the prey bdelloplast (and as HI cells), cellular components must be partitioned along the filament prior to division to ensure that they are faithfully segregated prior to synchronous septation. This contrasts the binary fission model of division, seen in most other bacteria, where conventional septation results in two daughter cells.

Pre-divisional partitioning is a process required for the organization of prokaryote cellular components, including chromosomes, plasmids, individual proteins [such as *E. coli* proteins UidR (transcriptional repressor), HisG (ATP phosphoribosyltransferase) and MalI (transcriptional repressor)], chemotaxis clusters and carboxysomes (Bignell and Thomas, 2001; Ringgaard et al., 2011, Roberts et al., 2012; Cho, 2015, Kuwada et al., 2015). The partitioning of DNA ensures that cell division does not occur across nucleoids, whilst the partitioning of proteins ensures that each daughter cell receives the prerequisite components for optimum fitness. Whilst some partitioning events may be stochastic, other events require active organization (Huh and Paulsson, 2011). A major checkpoint in the division cycle is the segregation of chromosomes, such that each progeny has a complete copy of the genome. In many bacterial species, such as *Vibrio* and *Caulobacter*, chromosome segregation is driven by a three component ParABS system which guides newly replicated chromosomes to bacterial cell poles, facilitating DNA segregation (Hui et al., 2010; Mierzejewska and Jagura-Burdzy, 2012, Espinosa et al., 2017). Here we identify a canonical ParAB system in *B. bacteriovorus* and show that it is required for efficient predatory growth.

In many bacteria, chromosomal segregation during cell division is controlled by the three element ParABS system via a ratchet diffusion model, as recently reviewed (Jindal and Emberly, 2019). ParB binds to centromere-like *parS* DNA sequences forming the ParBS nucleoprotein complex. ParA, an ATPase, will dimerize and bind DNA non-specifically in the presence of ATP. Chromosome segregation is facilitated by ParA-ATP interacting with ParBS complexes which activates the ATPase activity of ParA, causing it to dissociate from the chromosome, and for the ParBS complex to move. ParA-ADP can then be phosphorylated and will bind to DNA again after a delay. Therefore, as ParBS moves, it leaves an area of DNA behind it with no bound ParA-ATP, preventing the direction of ParBS movement from reversing. This allows the ParBS to move unidirectionally along a concentration gradient of bound ParA-ATP. This is a very attractive model but it is unclear how this system could maintain the position of multiple *B. bacterivorous* chromosomes along the growing filamentous cell prior to division.

In addition to *parA* and *parB*, there are several genes in the *B. bacteriovorus* genome that code for proteins that have been identified in other bacteria as key control elements for cell division. One of these is a *divIVA* homolog, hereafter referred to as *divIVA*_*Bd*_, which has been shown to encode a protein with a number of cell growth and septation related roles in Gram-positive bacteria, including septal site selection and chromosome segregation (Thomaides et al., 2001; Hammond et al., 2019). In Gram-negative bacteria, few studies have focused on the function of DivIVA homologs, which are typically limited to some Oligoflexia and Deltaproteobacteria (Akiyama et al., 2003), so we aimed to establish the function of DivIVA_*Bd*_ in *B. bacteriovorus* HD100, a Gram-negative predatory bacterium which grows inside prey.

Other bacterial genomes containing *divIVA* homologs can have non-conventional methods for septation. Mycobacteria are reported to have both symmetrical septation, producing two identical daughter cells, and asymmetric septation, where one daughter cell is significantly larger than the other (Kieser and Rubin, 2014; Vijay et al., 2014). In contrast, *Streptomyces* growth and division is characterized by polar (apical) growth of branched hyphae and the dispersion of spores, much like fungi (Flardh et al., 2012). The background of other proteins in addition to DivIVA (such as Noc and MinCD which are involved in the co-ordination of division site), can vary between Gram-positive bacteria so there is clearly no “one size fits all” scenario (Monahan et al., 2014; Hammond et al., 2019). In addition, in Streptococci and other bacteria, which like *B. bacteriovorus* lack MinCD, it was noted that post translational modifications of the DivIVA protein can also occur, and in Streptococci that a conserved alanine residue A78 is important for mediating DivIVA protein-protein interactions, suggesting a different regulatory network for septation control (Fadda et al., 2007). In these and other Gram-positive bacterial systems, DivIVA has been at least partially characterized as having varied roles, from regulating septation to polar cell wall growth (Hempel et al., 2008; Kang et al., 2008, Flardh et al., 2012; Ginda et al., 2013, Hammond et al., 2019). As apical growth is seen for the *B. bacteriovorus* filament, we postulated that DivIVA may be involved in this growth mode (Fenton et al., 2010a).

In *Bacillus subtilis*, the coiled-coil DivIVA protein is localized to negatively curved membranes by an N-terminus membrane-binding domain (Lenarcic et al., 2009). This localization allows DivIVA to interact with partner proteins to facilitate septum formation at mid-cell and chromosome segregation (Edwards and Errington, 1997; Marston and Errington, 1999, Thomaides et al., 2001). In other species the DivIVA protein directs hyphal tip extension in *Streptomyces coelicolor* (Flärdh, 2003; Hempel et al., 2008) and regulates polar growth in *Corynebacterium glutamicum* and *Mycobacterium smegmatis* (Kang et al., 2008; Letek et al., 2008, Donovan and Bramkamp, 2014; Kieser and Rubin, 2014, Meniche et al., 2014). However, in the cyanobacterium *Synechococcus elongatus* the DivIVA homolog Cdv3 does not contain the conserved residues linked to negative curvature sensing (MacCready et al., 2017).

Previous work has shown DivIVA and ParA directly interact in *M. smegmatis* (Ginda et al., 2013; Ramirez et al., 2013), establishing a link between the functions of both proteins. In other bacteria, DivIVA interacts with MinD, a ParA-like ATPase that functions in division site selection, either directly, such as in *Listeria monocytogenes* (Kaval et al., 2014), or indirectly, such as in *B. subtilis* where the interaction occurs via MinJ (Patrick and Kearns, 2008; Van Baarle and Bramkamp, 2010, Eswaramoorthy et al., 2011). In the *M. smegmatis* model, DivIVA directs subpolar addition to the cell wall during division (Kang et al., 2008), suggesting there is coordination between cell elongation and chromosome segregation. Given these roles across bacteria, DivIVA is a strong candidate to provide this type of coordination in *B. bacteriovorus* where complex, multi-septa division occurs along a filamentous cell (Fenton et al., 2010a).

Bacterial chromosomes often also encode orphan ParA-like proteins, which are additional ParA homologs not encoded from a canonical *parAB* locus. These ParA-like proteins perform roles distinct from ParA, for example PldP determines division sites in *Corynebacterium glutamicum* (Donovan et al., 2010; Donovan and Bramkamp, 2014), a ParA/Soj-like protein in *M. tuberculosis* interacts with the MzF6 protein regulating cell growth (Ramirez et al., 2013), and PpfA is involved in partitioning cytoplasmic chemotaxis clusters in *Rhodobacter sphaeroides* (Thompson et al., 2006; Roberts et al., 2012). These orphan *parA*-like genes are often, but not exclusively, found within operons containing genes for the processes in which they are involved. For example, *parC* (partitioning of chemotaxis) genes have been identified in chemotaxis operons in numerous bacterial species (Ringgaard et al., 2011). In *Vibrio cholerae*, ParC is involved in partitioning chemotaxis proteins, thus playing a role in chemotaxis itself, and influencing swimming and swarming (Ringgaard et al., 2011). To date no ParA-like proteins have been reported to influence gliding motility, however, discrete cellular locations for gliding motor complexes, at different points along a cell axis, are required for gliding function and Par proteins could participate in their positioning.

Bacterial gliding is a process characterized by the non-flagellar movement of a single cell on a solid surface and has been previously observed in many bacterial species, including *B. bacteriovorus* (Spormann, 1999; Mendez et al., 2008, Lambert et al., 2011; Asada et al., 2012, Zhu and McBride, 2016). Gliding motility can be subdivided into two categories; social (S)-motility, surface movement using pilus retraction in *Myxococcus xanthus*; and adventurous (A)-gliding motility, characterized by the movement of individual cells (McBride and Zhu, 2013; Jakobczak et al., 2015, Zhu and McBride, 2016). *B. bacteriovorus* is known to exhibit a form of A-motility which uses gliding engines and is independent of pili (Lambert et al., 2011). Abolition of gliding (for example, in a diguanylyl cyclase (*dgcA*) mutant), renders the *Bdellovibrio* cells unable to glide out and exit a prey cell after lysis (Hobley et al., 2012a). Thus gliding may be particularly relevant when *Bdellovibrio* prey upon bacteria within biofilms.

The *B. bacteriovorus* HD100 genome contains two orphan *parA*-like genes (*bd1326* and *bd2331*), in addition to the typical *parAB* locus (*bd3906-5*). Both of these *parA*-like genes in *B. bacteriovorus* are located at loci where neighboring genes encode putative proteins with unknown functions, rather than a ParB homolog. A study into the early prey invasion ‘predatosome’ of *B. bacteriovorus* revealed that both the *parA*-like genes were up-regulated during HI filamentous growth, but not at the 30 min stage of prey invasion by flagellate predators in liquid cultures (Lambert et al., 2010). However, this work focused upon early invasion events, and not those at later time points. If these ParA-like proteins function during division, as canonical ParA does, then it is reasonable to expect them to be up-regulated later in the cell cycle once the bdelloplast is established at a time when the *B. bacteriovorus* filamentous cell is rapidly growing. If relevant to gliding motility, we would not expect to see this up-regulation when the cell is flagellate or sessile. Hereafter, the three ParA homologs of *B. bacteriovorus* will be referred to as ParA1 (Bd1326), ParA2 (Bd2331) and ParA3 (Bd3906), the latter which is co-expressed with the gene for ParB (Bd3905).

In this study, we demonstrate that DivIVA_*Bd*_ participates in septal positioning during predatory replication with resultant effects on progeny morphology. We postulate that its regulatory role may be linked to cell partitioning through interaction with a protein product of its co-transcribed neighboring gene and the canonical *parA3* gene-product. DivIVA may also interact with proteins that could signal nutritional or oxygenic status within the bdelloplast prior to predator septation. We also show by fluorescent tagging that the canonical ParA3 is associated with the cell division process and that the tag affects protein function and perturbs *B. bacteriovorus* cell length. We further demonstrate that two additional ParA homologs, ParA1 and ParA2, are expressed in *B. bacteriovorus*, with gene deletion and fluorescent localization assays revealing that both are non-essential, but that ParA2 contributes to gliding motility behavior.

These results prime further biochemical studies on the changing nature of the bdelloplast environment as *B. bacteriovorus* grow within it. We show here that cell division, morphological and movement behaviors of intra-bacterial *B. bacteriovorus* are responding to DivIVA_*Bd*_ or Par protein controls and that some of these may be sensing changes in bdelloplast biochemistry.

## Materials and Methods

### Bioinformatics

Gene and protein sequences for *B. bacteriovorus* HD100 were acquired from Xbase (Chaudhuri et al., 2008). Gene and protein homologs were found with the NCBI BLAST program suite (Altschul et al., 1990). Pairwise alignments were generated in EMBOSS Needle and multiple alignments in EMBOSS ClustalO (Rice et al., 2000; Sievers and Higgins, 2018). Alignments were visualized in ESPript 3.0 (Robert and Gouet, 2014). Statistics and graphs were processed in IBM SPSS Statistics for Windows, Version 25.0. Armonk, NY: IBM Corp. Protein trees were generated through MEGA-X version 10.0.05 using the Maximum Likelihood method, with 1000 bootstraps (Kumar et al., 2018) and visualized in FigTree version 1.4.4^1^.

### Bacterial Strain Growth

*B. bacteriovorus* HD100 was used throughout and maintained in Ca/HEPES buffer preying upon *E. coli* S17-1 prey as previously described (Supplementary Tables S1A,B) (Lambert et al., 2006, 2016). Host independent *B. bacteriovorus* strains were grown as previously described (Lambert and Sockett, 2013). Prey were grown in YT broth for 16 h at 37 °C with shaking at 200 rpm (Supplementary Table S1C).

### Reverse Transcription PCR

RNA extraction and Reverse Transcriptase PCR (RT-PCR) assays were performed as previously described (Lambert et al., 2006, 2016). Using a Promega SV total RNA isolation kit, total RNA was extracted from samples taken throughout the time course. RT-PCR assays were performed using the Qiagen One-step RT-PCR kit. Reaction conditions are as follows: One cycle of 50 °C for 30 min then 95 °C for 15 min, followed by 25-30 cycles of 94 °C for 1 min, 48 °C for 1 min, 72 °C for 2 min, and then a 10 min extension at 72 °C after the 25-30 cycles, and ultimately held at 4 °C prior to gel analysis of products. Primers used for amplification are listed in Supplementary Table S2A. Primers used specifically for operon walking are listed in Supplementary Table S2B.

### Cloning Fluorescently Tagged and Deletion Constructs

Fluorescent constructs were made using previously described methods (Fenton et al., 2010b). Genes were cloned into a vector by removal of the stop codon such that the C-terminus of the gene product fused with the mCherry, or mTFP gene. This was then subcloned into the vector pK18*mobsac*B and then introduced into the *B. bacteriovorus* via a single crossover event at the 5’ end of the gene, such that the ORF-tag is transcribed from the endogenous promoter in a merodiploid strain. To illuminate the cytoplasm of the *B. bacteriovorus* cells within prey, we used a previously published strain with full gene replacement of cytoplasmic marker protein Bd0064 with Bd0064mCerulean (Raghunathan et al., 2019). Genomic deletions of specific *B. bacteriovorus* genes were made using methods previously described (Capeness et al., 2013; Lambert and Sockett, 2013, Lambert et al., 2016). Primers used for amplification of genes are listed in Supplementary Tables S2C,D.

### Fluorescent and Phase Contrast Microscopy

Microscopy images were acquired using a Nikon Eclipse Ti-E widefield inverted microscope equipped with an Andor Neo sCMOS camera, as previously described (Kuru et al., 2017). Semi synchronous predatory prey lysate cultures for time course microscopy through the predatory cycle were prepared as previously described (Lambert and Sockett, 2013). Images were processed in either SimplePCI software (HCImage.com) or FIJI (ImageJ) (Schneider et al., 2012).

### Gliding Motility Assays

Timelapse video microscopy was used to take images of *B. bacteriovorus* HD100 strains immobilized on 1% agarose/CaHEPES (Lambert et al., 2011). Single cells were selected by generating random regions of interest (ROIs) containing approximately 5–10 cells in each field of view. Following the cells through each frame allowed the time at which each cell started gliding to be observed. After an individual cell had been gliding for one hour (allowing an establishment period), the number of direction changes (reversals) was counted manually and recorded. To determine the gliding status and position of any fluorescent foci, cells were immobilized as above. After 400 min of incubation (a time chosen to allow prolonged incubation on a surface, and for gliding motility to commence), images were acquired every 15 min for an hour and analyzed in SimplePCI.

### Predation Efficiency Assays

Predatorily grown (prey/host dependent HD) *B. bacteriovorus* strains containing fluorescent tags were assayed against a *fliC1* merodiploid which served as an antibiotic marked “wild type” equivalent control. 50 ml predatory cultures were grown and filtered through a 0.45 μm filter to remove any remaining prey cells, these were matched by protein concentration using a Lowry assay and subsequently enumerated to confirm the number of *B. bacteriovorus* added. To serve as bacterial prey, a 50 ml culture of luminescent S17-1 pCL100, which encodes *luxCDABE*, was also grown under standard conditions. A 1.5 ml sample of each *B. bacteriovorus* strain was heat-treated at 105°C for 5 min and allowed to cool to room temperature, generating the ‘heat killed’ control cells. Equivalent amounts of each Lowry-matched *B. bacteriovorus* strain was made up to 64 μl with the heat-killed *B. bacteriovorus* preparation (64, 32, 16 and 8 μl live *B. bacteriovorus* plus 0, 32, 48 or 56 μl heat-killed cells). Predatory cells were mixed with 200 μl of *E. coli* S17-1 pCL100 and diluted to an OD_600_ of 1.0 in CaHEPES, in a 96-well microtiter plate. Control wells had 64 μl heat-killed *B. bacteriovorus* only for each strain. The plates were then covered with Breathe-Easy membrane. The reduction in luminescence due to the killing of the *E. coli* S17-1 pCL100 prey cells by *B. bacteriovorus* was measured over time using a BMG FluoStar microplate reader.

### Cell Morphology Measurements Using MicrobeJ and Detection of Fluorescent Foci of DivIVA

After image acquisition, cell measurements were generated using MicrobeJ, a plugin for the FIJI software, as described previously in Kuru et al., 2017 (Schindelin et al., 2012; Ducret et al., 2016, Kuru et al., 2017). To detect fluorescent foci of DivIVA-mCherry; the rounded, invaded, *E. coli* prey cells (bdelloplasts) were detected in the phase channel by defining circularity as 0.96-max and length as 1-max, with all other parameters as default. *B. bacteriovorus* cells (with cytoplasmic Bd0064-mCerulean) were detected by the medial axis method in the mCerulean channel as maxima 1, by defining area as 0.15-max, with all other parameters as default. They were associated with the bdelloplasts with a tolerance of 0.1. Fluorescent foci of DivIVA-mCherry were detected as maxima 2 in the red channel by the “fit shape to circle” method by defining area as 0-0.25, with all other parameters as default. These were associated to maxima 1 with a tolerance of 0.1. Manual inspection of the analyzed images confirmed that the vast majority of cells were correctly assigned. In cases where bdelloplasts appeared to be infected by two *Bdellovibrio*, these were manually removed from the analysis. The shape measurements including the angularity, area, aspect ratio, circularity, curvature, length, roundness, sinuosity, solidity and width were measured for each type of cell.

### Pairwise Bacterial Two Hybrid and Liquid β-Galactosidase Assays

Pairwise Bacterial Two Hybrid (BTH) assays were performed to test for interactions between DivIVA_*Bd*_, ParA3 and other *B. bacteriovorus* proteins using protocols previously described (Battesti and Bouveret, 2012). Liquid β-galactosidase assays were performed using the single-step protocol (Schaefer et al., 2016). Primers used for gene amplification are listed in Supplementary Table S2E.

### *B. bacteriovorus* HD100 Bacterial Two Hybrid Library Construction and Assay

A *B. bacteriovorus* HD100 BTH library was constructed following methods, adapted from those previously described (Handford et al., 2009; Houot et al., 2012). Briefly, the HD100 genome was extracted, using the Sigma-Aldrich GenElute Bacterial Genomic DNA Kit, and restriction-digested into fragments between 500 bp and 2000 bp which were extracted via the Sigma-Aldrich GenElute Gel Extraction Kit, according to manufacturer’s instructions. This was then ligated into four plasmids; pUT18, pUT18C and pUT18 + 1, where + 1 denotes an additional nucleotide added in the linker region to account for the frame of the genome fragments. The ligations were transformed into *E. coli* DH5α, and the resulting transformants were pooled. Plasmids were extracted using the Sigma-Aldrich GenElute Plasmid Miniprep Kit, resulting in a library of mixed genomic fragments for each of the plasmids.

To perform the assay, a pKT25 plasmid containing the bait gene was transformed into the *E. coli* BTH101 strain, which was made chemically competent. These were transformed with 1μl of library plasmids. Incubation was for 48 h at 29^*o*^C on MacConkey agar supplemented with 50 mg/ml Ampicillin, 25 mg/ml Kanamycin, 40 mg/ml X-gal and 20 mg/ml IPTG. Blue colonies were grown in high salt Mu media and the plasmids, bait and library were extracted via miniprep. Competent *E. coli* DH5α were then transformed with both plasmids and cured for the pKT25 plasmid. The library plasmid was then extracted. This was retested with the BTH pairwise protocol against the bait plasmid to confirm transformants remain blue on the supplemented MacConkey agar. These were then sequenced to determine the interacting protein fragments encoded in the library fragments. Once the genes in the fragments were identified, full length gene copies were cloned into pUT18C and pKT25 and used again in the pairwise assay above.

## Results

### *B. bacteriovorus* DivIVA_*Bd*_ Is Encoded on a Four Gene Operon

**FIGURE 1:**
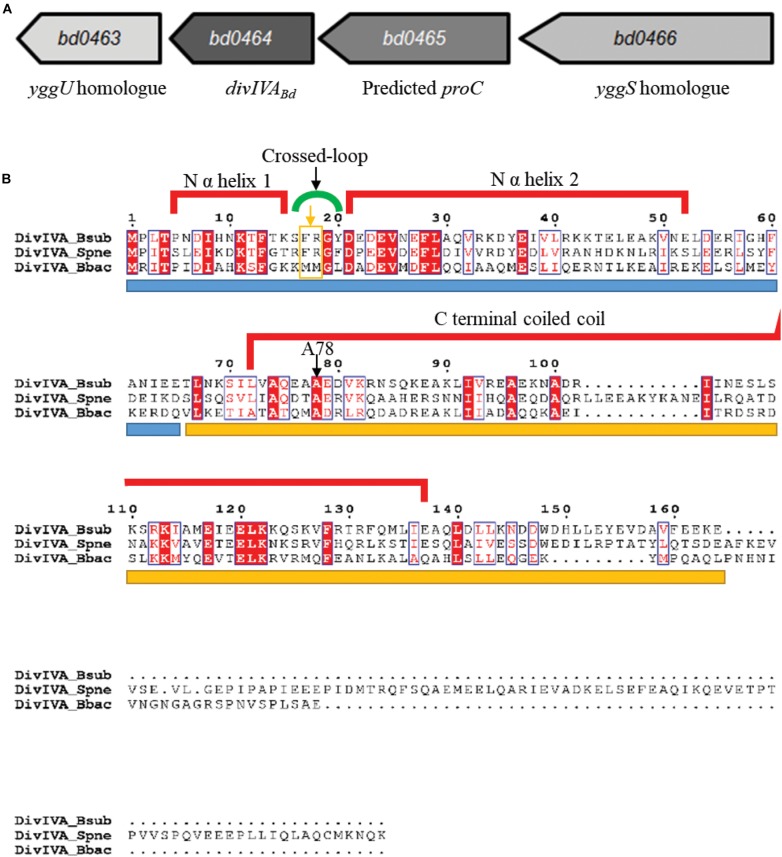
Genomic context of the *B. bacteriovorus divIVA*_*Bd*_ gene and protein alignment of DivIVA_*Bd*_ with Gram-positive DivIVA homologs. **(A)** Map of the *B. bacteriovorus divIVA* gene and neighboring region. The *divIVA*_*Bd*_ gene is downstream of genes encoding a predicted *yggS* homolog (Bd0466 accession: NP_967454.1) and pyrroline-5-carboxylate reductase (Bd0465 accession: NP_967453.1) and upstream of a gene encoding an *YggU* homolog (Bd0463 accession: NP_967451.1). **(B)** Alignment of DivIVA_*Bb*_ with DivIVA homologs in *Streptococcus pneumoniae* (DivIVA_*Spn*_; accession: AAC95445.1) and *Bacillus subtilis* (DivIVA_*Bsub*_; accession: P71021.1). Sequences are colored and annotated based on the DivIVA_*Bsub*_ crystal structure. The blue bar under the sequences indicates the N terminal domain and the yellow bar, the C terminal domain. The A78 residue, conserved in DivIVA_*Bd*_, was shown to be important for DivIVA function in *B. subtilis*, and an A78T substitution in *S. pneumoniae* DivIVA disturbed protein localization and perturbed interactions with FtsK and late-stage divisome components. Residues F17 and R18 are highlighted by an orange box and arrow underneath the green cross-link indicator.

DivIVA proteins are usually found in Gram-positive genomes. Despite this, the Gram-negative *B. bacteriovorus* genome contains a DivIVA homolog encoded by *bd0464* and referred to as DivIVA_*Bd*_ (Figure 1A). Comparing this protein to features of DivIVA in *Bacillus subtilis* (detailed in Supplementary Information S1) revealed that DivIVA_*Bd*_ can likely dimerize, but substitutions in R18 suggest that it cannot inherently sense negative curvature, likely requiring other protein interactions to localize to curved bacterial cell poles.

DivIVA homologs of Gram-positive bacteria are typically encoded downstream of a cell division and cell wall (*dcw*) cluster and the *ftsZ* gene (Ayala et al., 1994; Cha and Stewart, 1997, Massidda et al., 1998; Ramirez-Arcos et al., 2005). Although *B. bacteriovorus* does have a *dcw* cluster, *divIVA*_*Bd*_ (*bd0464*, accession number: NP_967452.1) is not found near this region (Figure 1A), rather it is downstream of a *yggS* homolog, *bd0466*, which encodes a protein similar to the N-terminal barrel domain of an alanine racemase (LeMagueres et al., 2005; Ito et al., 2013). In *E. coli*, this domain binds the coenzyme Pyridoxal Phosphate (PLP), with the required residues conserved in Bd0466 (Ito et al., 2013). Recently, the function of YggS has been studied in other bacteria, implicating it in the regulation of PLP and biosynthesis of amino acids isoleucine and valine (Ito et al., 2013, 2016, Prunetti et al., 2016; Ito et al., 2019). In species such as *Bacillus subtilis*, *Staphylococcus aureus*, and *Streptococcus pneumoniae*, a *yggS* protein is also found upstream of the *divIVA* homolog.

Between *bd0466* and *divIVA*_*Bd*_ is the gene *bd0465*, which encodes a pyrroline-5-carboxylate reductase, ProC, homolog (Figure 1A). ProC proteins catalyze the final step in the biosynthesis pathway that converts glutamate to proline (Nocek et al., 2005).

Alignment of the *B. bacteriovorus* DivIVA_*Bd*_ with DivIVA homologs of *Streptococcus pneumoniae* and *Bacillus subtilis* (Figure 1B) showed that the N-terminal domain and coiled-coil domains [confirmed using Multicoil prediction (Wolf et al., 1997)] are conserved between the proteins. A NEEDLE global alignment showed that DivIVA_*Bd*_ and DivIVA_*Bsub*_ share 24.6% protein identity and 47.5% protein similarity, with differences predominantly at the C-terminus. This is typical of DivIVA proteins, as the C-terminus of different DivIVA homologs tends to show greater variability (Tavares et al., 2008).

### DivIVA_*Bd*_-mCherry Is Localized at the Poles of *B. bacteriovorus* Attack Phase Cells and Growing Filaments

**FIGURE 2:**
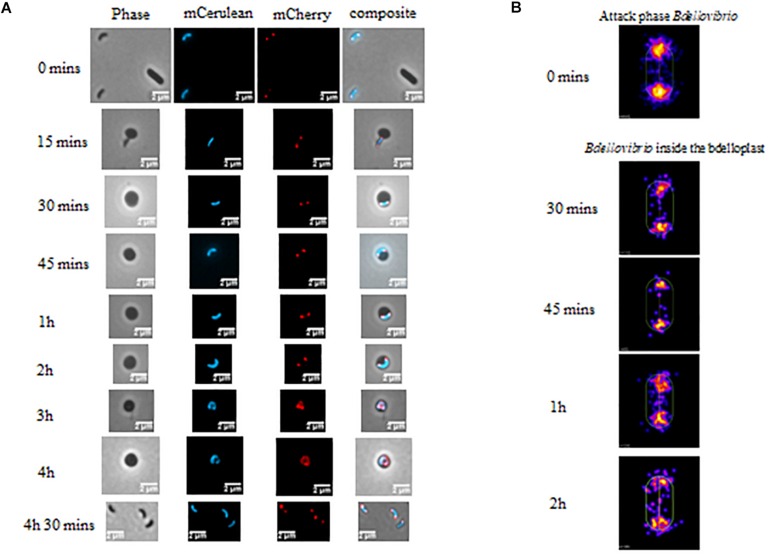
**(A)** Phase and epifluorescence microscopy displaying the location of DivIVA_*Bd*_ tagged with mCherry. Localization was observed during invasion and growth within *E. coli* prey cells. The *Bdellovibrio* cytoplasm is constitutively fluorescent with Bd0064-mCerulean to visualize the cell within the bdelloplast. DivIVA-mCherry localizes to the poles of the *Bdellovibrio*, except at the 3–4 h timepoints, where it is less clear due to the growing filament extending and twisting in three dimensions as it elongates (see Supplementary Figure S1). Images are representative of three biological repeats. Scale bars are 2 μm. **(B)** Cellular position maps of fluorescent foci of DivIVA-mCherry detected by MicrobeJ. All of the cells detected (including free swimming attack phase cells) were measured at time 0, but only cells within bdelloplasts were measured at the other timepoints. Data are pooled from three independent experiments (*N* values of cells at each timepoint: T0- 845, T30- 110, T45–65, T1h- 226, and T2h- 120).

As DivIVA_*Bd*_ is lacking residues shown to be essential for negative curvature sensing, F17 and R18 (highlighted in Figure 1B), we wanted to assess its localization in host-dependent *B. bacteriovorus* cells. The protein was labeled with C-terminal mCherry fluorescent protein and attack-phase *B. bacteriovorus* cells were analyzed by fluorescent microscopy. This revealed DivIVA_*Bd*_-mCherry localizes to both poles of host-dependent *B. bacteriovorus* cells (Figure 2). This is consistent with the localization pattern of DivIVA homologs in *Bacillus* and relevant to the monopolar localization in Streptococci and *Mycobacterium.* (Marston et al., 1998; Fadda et al., 2007, Nguyen et al., 2007).

Fluorescent localization of DivIVA_*Bd*_-mCherry was then assessed at stages of intracellular growth. This showed that the fluorescently tagged protein was found at both poles of the growing *B. bacteriovorus* filament during predation for up to 2 h in the prey bdelloplast (Figure 2). At later timepoints (3–4 h post-invasion), the situation was less clear as the growing filament twists in three dimensions as it grows and divides in the bdelloplast. A mixture of some single or double foci and more diffuse DivIVA_*Bd*_-mCherry fluorescence was seen. This likely represents the DivIVA_*Bd*_-mCherry migrating to the newly forming poles of the *B. bacteriovorus* progeny (Figure 2 and further examples in Supplementary Figure S1).

To determine whether expression of DivIVA_*Bd*_-mCherry affected progeny number and the general predation rate, we conducted an assay of predation on luminescent prey (Lambert et al., 2003). This clearly showed that the predation rate of the DivIVA_*Bd*_-mCherry strain was not different to that of the FliC1 merodiploid (“wild-type” equivalent) control (Supplementary Figure S2A; Mann-Whitney test *p* = 0.995; n = 35). In addition, measuring intraperiplasmic growth area of the *B. bacteriovorus* (as total area of mCerulean fluorescence) showed no significant difference for this strain compared to wild-type (Supplementary Figure S2B), suggesting that the C-terminal mCherry tagging of DivIVA_*Bd*_ has no detrimental effect on growth.

### Cells Lacking DivIVA_*Bd*_ Show Morphological Changes in Attack-Phase *B. bacteriovorus* Progeny Cells

DivIVA is essential in *Streptomyces coelicolor*, *Enterococcus faecalis*, and *Mycobacterium smegmatis*, so we wanted to determine if this is also the case in *B. bacteriovorus* (Flärdh, 2003; Ramirez-Arcos et al., 2005, Kang et al., 2008). Deletion of *divIVA*_*Bd*_ was possible in predatory *B. bacteriovorus*, demonstrating that DivIVA_*Bd*_ is not essential for *B. bacteriovorus* predatory growth and division.

**FIGURE 3:**
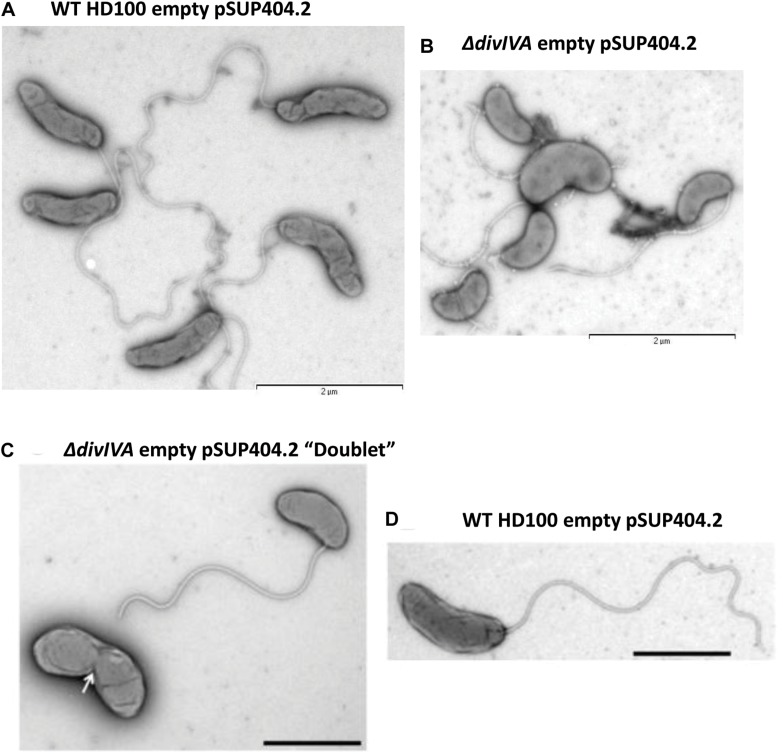
Morphological changes in the attack phase cells in *B. bacteriovorus*Δ*divIVA*_*Bd*_ deletion strain grown with *E. coli* S17-1. TEM images of **(A)** Wild-type HD100 (pSUP404.2) cells are long and slender, whilst Δ*divIVA*_*Bd*_ (pSUP404.2) cells **(B)** are shorter and wider. **(C)** A rare a doublet cell of Δ*divIVA*_*Bd*_ with pinched septum. **(D)** *B. bacteriovorus* HD100 wild-type attack phase cell. Cells were stained with 0.5% uranyl acetate. Scale bars are 2 μm.

**FIGURE 4:**
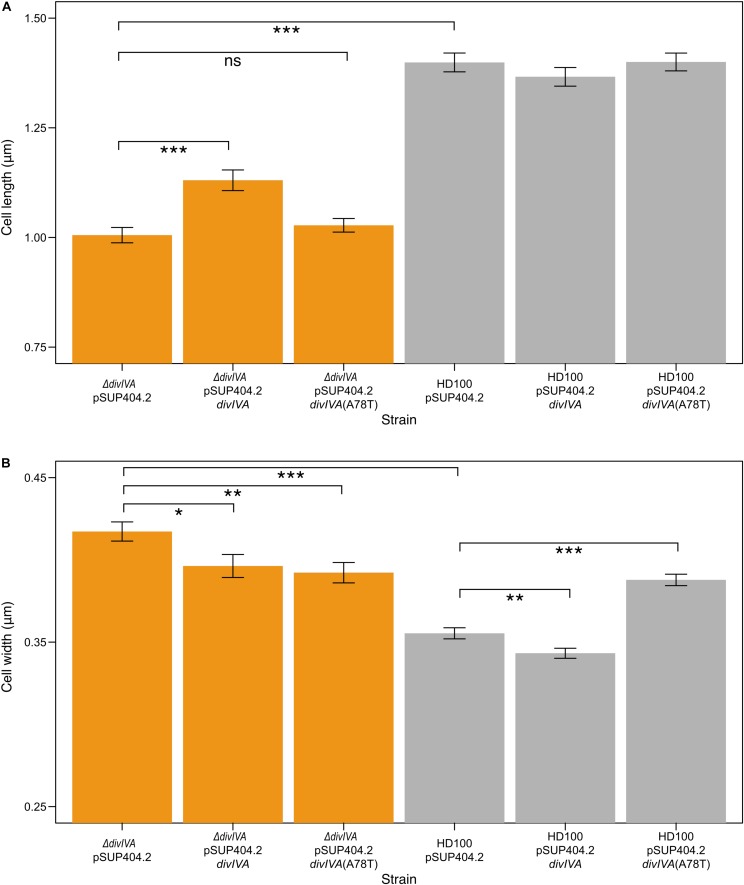
Δ*divIVA*_*Bd*_ strains are shorter and wider than wild type, and are partially restored through complementation. Bar charts showing the mean lengths **(A)** and widths **(B)** of *B. bacteriovorus* strains containing a pSUP404.2 plasmid either empty, or encoding DivIVA_*Bd*_, or a mutant DivIVA_*Bd*_(A78T). **Δ***divIVA*_*Bd*_ strains are significantly shorter (*P* < 0.001) than wild type, with partial restoration of length when *divIVA*_*Bd*_ is introduced on plasmid pSUP404.2 (*P* < 0.001). Average width of **Δ***divIVA*_*Bd*_ strains is significantly greater than wild type (*P* < 0.001) with partial restoration when complemented with *divIVA*_*Bd*_ (*P* < 0.05) or *divIVA*_*Bd*_(A78T) (*P* < 0.01). Width measurements show that wild type HD100 containing a plasmid with *divIVA*_*Bd*_ are thinner (*P* < 0.01), and wider with *divIVA*_*Bd*_(A78T) (*P* < 0.001), suggesting that *in trans* expression levels may perturb DivIVA function. *n* = 75 for each population and data are from three biological repeats (all significance calculated as *p ≤ 0.05, **p ≤ 0.01, ***p ≤ 0.001, using a *t*-test). Images were acquired through transmission electron microscopy and analyzed in SimplePCI.

Transmission electron microscopy of progeny (attack phase) cells recently emerged from bdelloplasts revealed that the *B. bacteriovorus* Δ*divIVA*_*Bd*_ strain had a morphological defect resulting in shorter, wider cells (Figure 3). Wild-type *B. bacteriovorus* HD100 pSUP404.2 (empty vector) cells had a mean length of 1.40 ± 0.04 μm and mean width of 0.36 ± 0.01 μm (n = 75). The Δ*divIVA*_*Bd*_ mutant had a shorter mean length of 1.01 ± 0.03 μm (p < 0.001; n = 75) and larger mean width of 0.42 ± 0.01 μm (p < 0.01; n = 75) (Figure 4). Additionally, rare doublet cells were observed in the attack phase population (at less than 1%) (Figure 3C compared to D). These appeared to have an incompletely divided septum, which pinched in between the non-divided cells, resulting in a single longer cell. These morphological defects were also apparent through analysis of attack phase cell images by phase contrast microscopy and analyzed using the MicrobeJ plugin (Ducret et al., 2016). This analysis showed wild type cells to have a mean length of 1.34 ± 0.06 μm and width of 0.47 ± 0.004 μm (n = 2,302). In comparison, the Δ*divIVA*_*Bd*_ strain was shorter, 1.01 ± 0.004 μm (*p* < 0.001, n = 2796), and marginally wider, 0.50 ± 0.004 μm (*p* < 0.001, n = 2796).

To confirm these effects on morphology were due to the lack of DivIVA_*Bd*_, we introduced a wild type *divIVA*_*Bd*_ gene expressed *in trans* on plasmid pSUP404.2 into the Δ*divIVA*_*Bd*_ strain. When compared to Δ*divIVA*_*Bd*_ containing an empty control vector this partially restored cell length and width of attack phase cells when imaged by TEM (*p* < 0.001, n = 75; Figure 4). This confirms that the deletion strain phenotype is attributable to the loss of a functional *divIVA*_*Bd*_ locus. Further, there were no observed doublet cells in the complemented strains. To investigate this link between cell division and *divVIA*_*bd*_ further, we complemented these strains with a *divIVA_*Bd*_-*A78T mutated copy of the gene (Figure 4). In *Streptococcus pneumoniae, divIVA*-A78T mutants had impaired cell division but normal chromosome segregation (Fadda et al., 2007). The Δ*divIVA*_*Bd*_ strain complemented with *divIVA-*A78T had a mean length of 1.03 ± 0.13 μm, which was not significantly different to Δ*divIVA*_*Bd*_ with an empty pSUP404.2 plasmid (n = 75). However, its width, 0.39 ± 0.05 μm, was partially restored and significantly lower than Δ*divIVA*_*Bd*_ (*p* < 0.01, n = 75).

### *B. bacteriovorus divIVA* Is Expressed Throughout Growth and Is Co-transcribed With *bd0465* and *bd0466*

**FIGURE 5:**
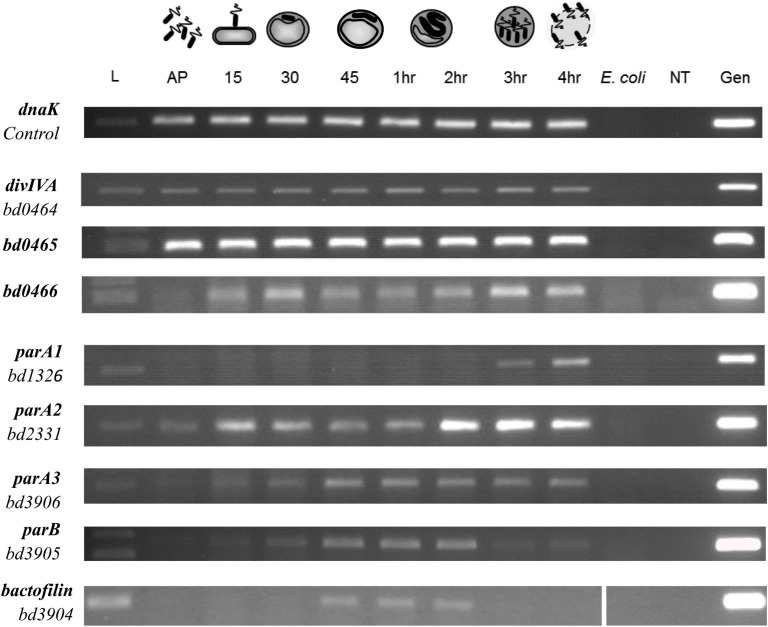
Expression pattern of *B. bacteriovorus* HD100 *parA-*like genes, *parB*, *bactofilin, divIVA_*Bd*_*, and its upstream genomic neighbors *bd0465* and *bd0466*. Expression was assessed alongside control gene *dnaK* throughout the predatory life cycle using RT-PCR with transcript specific primers. RNA was prepared from identical volumes of infection cultures with *B. bacteriovorus* HD100 predator and *E. coli* S17-1 prey at time-points throughout the predatory life cycle. AP: RNA from attack-phase *B. bacteriovorus*, 15–45: RNA from 15–45 min time-points through the predatory cycle, 1–4 h: RNA from 1–4 h through the time-course, *E. coli*: *E. coli* S17-1 only template RNA, -ve: no template negative control, Gen: *B. bacteriovorus* HD100 genomic DNA template as positive control. Images are representative of two biological repeats.

Using a semi-quantitative Reverse Transcription PCR (RT-PCR) approach, transcription of the *divIVA*_*Bd*_ gene and the neighboring genes *bd0465* and *bd0466* (Figure 1) were assessed across the *B. bacteriovorus* predatory cycle (Figure 5). We hypothesized that transcription of the gene cluster containing *divIVA*_*Bd*_ might be up-regulated when the gene products were required for function. This approach showed that all three genes were constitutively expressed throughout the host dependent cycle (Figure 5).

To validate the hypothesis that *divIVA*_*Bd*_ is expressed in an operon with its neighboring genes *bd0465* and *bd0466* (Figure 1), we wanted to determine whether these genes were co-transcribed. RNA-seq analysis suggested that transcription occurs across this cluster of genes, but that *divIVA*_*Bd*_ has its own promoter, as it had a higher transcription level than *bd0465* and *bd0466* (Reads Per Kilobase Million for *bd0466* = 9.56, *bd0465* = 121.04, *divIVA_Bd_* = 1554.641) (Capeness et al., 2013). Promoter walking confirmed that *bd0465* and *divIVA*_*Bd*_ were co-transcribed, as well as showing that *divIVA*_*Bd*_ has its own promoter. An RT-PCR assay confirmed that *bd0466* and *bd0465* were also co-transcribed (Supplementary Figure S3).

#### Bd0465-mCherry and Bd0466-mTFP Are Localized in the Cytoplasm and Pairwise BTH Testing Shows Bd0465 Interacts With DivIVA_*Bd*_, Bd0466, and ParA3

Given that DivIVA_*Bd*_ fluorescence was seen at polar foci (Figure 2), we assessed the localization of Bd0465 and Bd0466 through fluorescent microscopy. To do so Bd0465-mCherry and Bd0466-mTFP constructs were conjugated into wild type *B. bacteriovorus* HD100. In contrast to the polar foci of DivIVA_*Bd*_-mCherry, both tagged proteins were ubiquitously expressed in the cytoplasm in attack phase cells. Images were taken throughout the predatory life cycle, but the fluorescence was too faint to accurately determine localization when the *B. bacteriovorus* was within the bdelloplast (Supplementary Figure S4).

**FIGURE 6:**
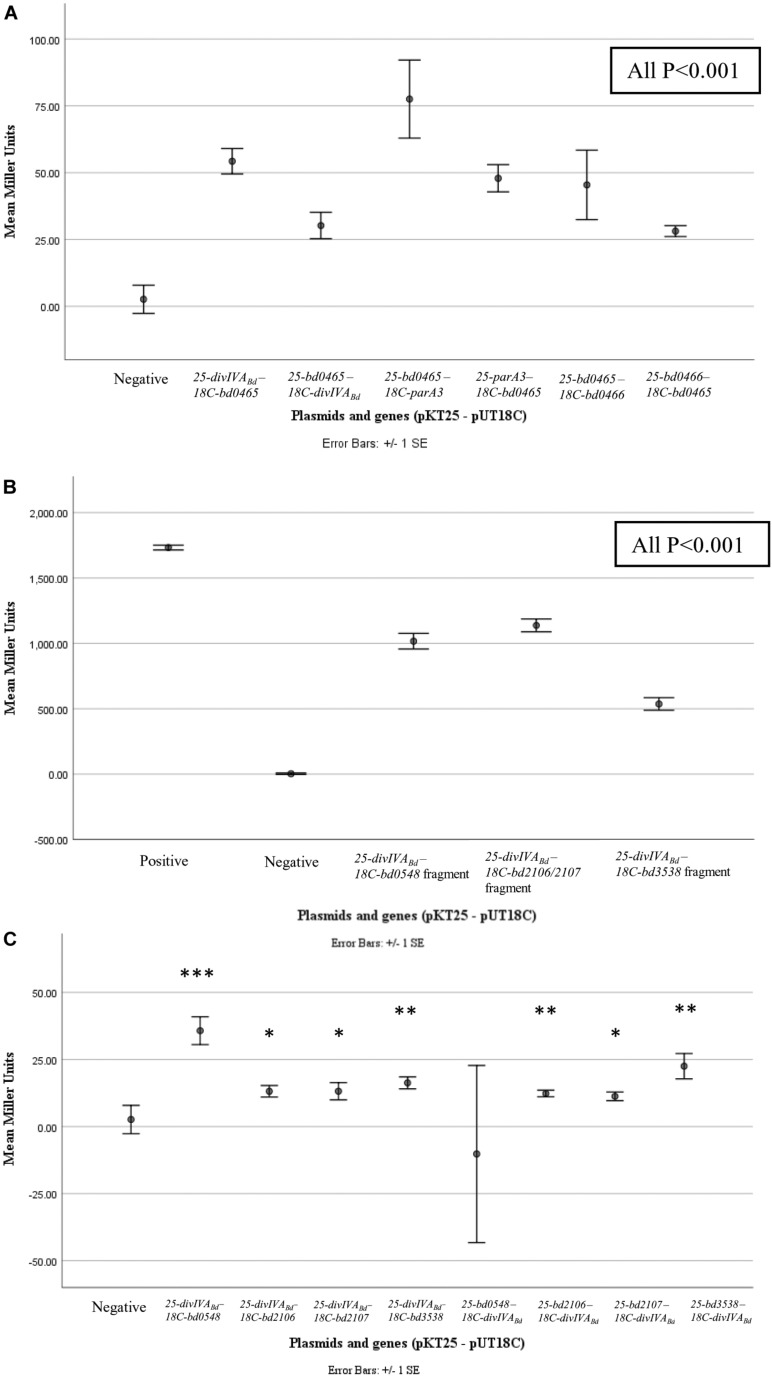
β-galactosidase assay results for pairwise and library screening bacterial two hybrid interactions. Points represent average Miller Units of cotransformants (*n* = 16), error bar represent standard error. **(A)** shows significant interactions between DivIVA_*Bd*_ (Bd0464) and Bd0465, Bd0465 and ParA3 (Bd3906), and Bd0465 and Bd0466 (all *P* < 0.001) when compared to the negative control. **(B)** shows significant interactions between protein fragments encoded from BTH library plasmids, specifically partial proteins of Bd0548, Bd2106, Bd2107, and Bd3538, with DivIVA_*Bd*_ plasmids (all *P* < 0.001). **(C)** shows results for interactions between DivIVA_*Bd*_ and full length proteins Bd0548, Bd2106, Bd2107, and Bd3538 (***P < 0.001, **P < 0.01, and *P < 0.05). Data are from two biological repeats. Individual data points are presented in Supplementary Figure S5.

To determine if DivIVA_*Bd*_ interacts with its co-transcribed neighboring proteins we used the Bacterial Two Hybrid assay. In addition, we also tested a selection of known *B. bacteriovorus* cell division related proteins. Bacterial Two Hybrid (BTH) assays were performed in a pairwise manner between candidate proteins. For confirmation and quantification, the positive interactions were subjected to β-galactosidase assays. These assays showed significant results (*p* < 0.001) for interactions between Bd0465-DivIVA_*Bd*_, Bd0465-Bd0466 and Bd0465-ParA3 (Figure 6 and Supplementary Figure S5). No interaction was detected between DivIVA_*Bd*_-Bd0463, and for DivIVA_*Bd*_ with ParA1, ParA2 or ParA3. We later turned to analyze Par protein function (see below) as there is a potential three-way interaction between Bd0465-DivIVA_*Bd*_, and Bd0465-ParA3. Further potential DivVIA_*bd*_ interacting partners were sought by an unbiased BTH library screening method (Supplementary Information S2).

### *B. bacteriovorus* Has Three Genes With Homology to *Bacillus subtilis soj* (*parA*)

Our cell morphology phenotype and fluorescence-localization experiments suggest that DivIVA_*Bd*_ may have an ancillary role in co-ordinating division with cell growth (Figures 2–4). Its link to canonical ParA3 (*bd3906*) through three-way (Bd0465-DivIVA_*Bd*_, and Bd0465-ParA3) BTH protein interactions (Figure 6) then led us to investigate the role of chromosome-partitioning genes. Three genes in the *B. bacteriovorus* HD100 genome encode proteins with similarity to Soj, the ParA ortholog in *B. subtilis*. The product of *bd3906* shares 57.71% protein identity with Soj, whilst the other predicted proteins of ParA1 and ParA2 share 27.94% and 35.48% identity, respectively (Supplementary Figure S6). Gene *parA3* is located upstream of a *parB* homolog, in the arrangement typically seen at the *parAB* locus. Attempts at deleting *parA3* were unsuccessful, despite screening many host dependent and host independent exconjugants (Chang et al., 2011), suggesting that ParA3 may be essential in *B. bacteriovorus*. The products of the genes neighboring *parA1* and *parA2* (*bd1327* and *bd2329*) do not show any strong homology to ParB, or to any other proteins of known function, and show only weak homology to each other (12% sequence identity). Adjacent to *parA1* is a *bolA*-like gene (*bd1328*). BolA is a transcription factor involved in the regulation of penicillin-binding proteins PBP5 and PBP6, and of MreB (Guinote et al., 2011; Singh and Montgomery, 2014).

### Expression of *parA*-like Genes Peaks Toward the End of the *B. bacteriovorus* Predatory Cycle, and all Three Genes Have Differing Transcription Patterns

RT-PCR was carried out on all three *parA*-like genes in *B. bacteriovorus* (Figure 5). This demonstrated that expression of *parA1* peaks at 3–4 h post infection at the very late stage of *B. bacteriovorus* filamentous cell division and prey exit. Expression of *parA2* peaks at 2–4 h (with a peak at 15 min post-infection), whilst the canonical *parA3* shows an increase in expression up to 45 min post-infection, then exhibiting consistent expression until the end of the predatory cycle. These later *parA2* and *parA3* profiles are consistent with stable expression across the *B. bacteriovorus* filamentous growth phase (Figure 5).

RT-PCR analysis also suggested that all three *parA* genes are encoded within operons (Supplementary Figure S7). Co-expression of *parA1*, *bd1327*, and *bd1328* (encoding a BolA homolog) was observed at the 3 h time point, whilst *parA2* was found to be co-transcribed with *bd2329* at 3 h post-infection. Co-transcription of *parA3, bd3905* (*parB*) and *bd3904* (encoding a bactofilin homolog) was observed at 1 h post-infection.

### Fluorescent Tagging of the Canonical ParA3 in *B. bacteriovorus* Creates Some Longer Attack-Phase Cells and Slows the Overall Rate of Predation

**FIGURE 7:**
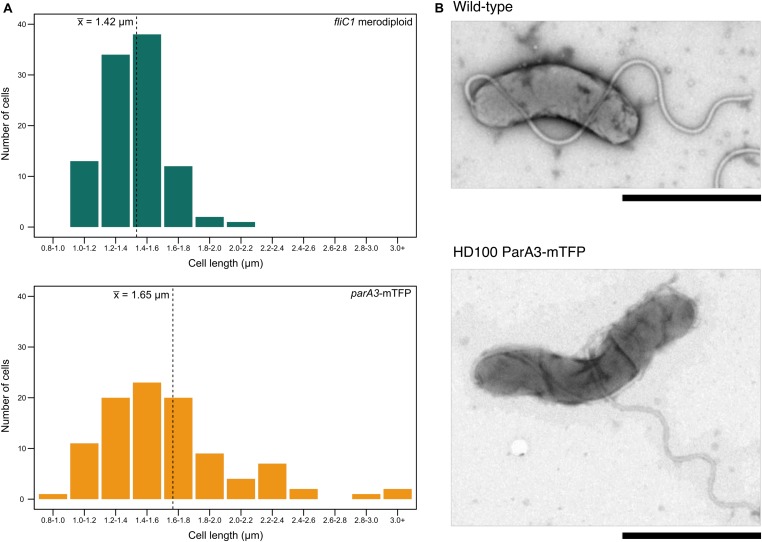
Cell length distributions for attack phase cells of wild type and ParA3-mTFP strains. **(A)** Distribution of cell lengths for *B. bacteriovorus fliC1* merodiploid strain (“wild type” equivalent, green) and HD100 ParA3-mTFP (yellow) populations (*n* = 100). The mean is denoted by the dotted line. Representative TEM images of both populations are shown in **(B)**. Scale bars are 1 μm and data are from three biological repeats.

**FIGURE 8:**
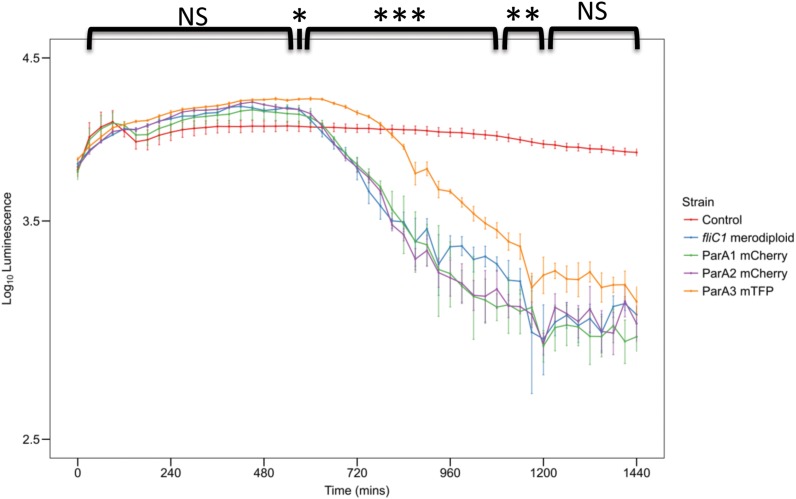
Plot of prey luminescence decrease over time comparing the predation rates of *B. bacteriovorus* cells expressing C terminally, fluorescently tagged ParA1, ParA2, or ParA3. Predation rate is measured as the drop in luminescence from the prey as they are preyed upon by *Bdellovibrio*, compared to wild type equivalent strain *B. bacteriovorus* FliC1 merodiploid (blue) (*n* = 12 technical replicates per strain for three biological repeats). Only the tagged ParA3 strain (orange) is delayed in causing a logarithmic drop in luminescence (which represents killing of the luminescent prey) compared to the other 3 strains. Control: *E. coli* prey only. ***p < 0.001, **p < 0.01, *p < 0.05, and NS not significant by Mann-Whitney U test.

When *parA3*(*bd3906*) was originally tagged with mTFP, the mean length of attack phase cells increased compared to wild type (16.7% increase) suggesting that the tagged protein was only partly functional (Figure 7). Quantification and analysis of electron microscopy images showed the distribution of lengths, with a higher number of very long cells observed in the *parA3-mTFP* population. This was repeated with ParA3-mCherry (to be comparable with the other two ParA1 and 2 strains tagged with mCherry) and a similar small excess of very long cells was detected in the population. Next we measured the predation rate of *B. bacteriovorus* strains containing tagged *parA1, parA2*, and *parA3* (Figure 8). These data showed only the strains containing the *parA3-mTFP* had a significantly slower overall predatory growth (Figure 8). To see if tagging *parA3* affected nucleoid position and copy number, attack phase cells were stained with DAPI. Through fluorescent microscopy and image analysis in SimplePCI software, lengths of nucleoid and cell were measured and the ratios compared. This ratio was significantly lower for *bd3906-mTFP* (mean nucleoid length/cell length ratio of 0.55 ± 0.15 μm) than wild type (0.63 ± 0.1 μm. P < 0.05) suggesting a constant nucleoid length which is independent of cell length (Supplementary Figure S8). However, there was no significant difference between the proportion of anucleate cells in wild type (0/804 cells) and *Bd3906-mTFP* cells (1/702 cells) (Fisher’s exact test p = 0.466).

### ParA2 May Have a Role in Gliding Motility

Initial experiments on deletions of *parA1* and *parA2* implicate a role for ParA2 in gliding motility behavior, including the number of reversals of gliding direction after the first hour on a solid surface and the number of cells initiating productive (non-rapidly reversing) gliding behavior after that hour-long adaptation period (Supplementary Information S3 and Supplementary Figures S9–S11). However, as both strains could be constructed in predatory cultures, neither was required for predatory growth and division. Using the top homologous sequences from pBLAST, protein trees were constructed to show the relationship between the three ParA homologs in *B. bacteriovorus* and proteins of other prokaryotes (Supplementary Figure S12 and Supplementary Information S4).

## Discussion

The constitutive expression of DivIVA_*Bd*_ and its bi-polar localization throughout 2 h of predatory growth until diffusion around the time point of septation and new cell pole specification, suggested that DivIVA_*Bd*_ coordinates important processes from the cell poles during predatory growth.

Morphological changes in the *divIVA* deletion strain may be a consequence of aberrant cell wall regulation and changes in septal positioning. Consequent adaptation to conserve cell volume could cause the short-wider morphology of *B. bacteriovorus* progeny that had a *divIVA*_*Bd*_ deletion. To determine whether this is the case, septal and sub-polar peptidoglycan labeling would be required, which is beyond the scope of this study. We note that in *M. smegmatis* incorporation of new sub-polar peptidoglycan is coordinated by a DivIVA homolog (Kang et al., 2008; Ginda et al., 2013).

Interestingly, DivIVA_*Bd*_-mCherry was present at both poles in newly formed *B. bacteriovorus* progeny, suggesting possible inheritance of at least one focus of the protein rather than *de novo* synthesis of both foci in progeny cells. Due to the constraints of *B. bacteriovorus* growing in 3D inside another cell, as a long twisting filament, prior to and during septation it is hard to tell when the polar DivIVA_*Bd*_ migrates to the septa. However, some diffusion, but still some single foci, of DivIVA_*Bd*_-mCherry were observed at the three hour time point of the predatory cycle (Figure 2 and Supplementary Figure S1). This contrasts with the bipolar fluorescence seen at two hours, suggesting a transition in DivIVA position from filament growth to septation, facilitating progeny inheritance of the protein. In *B. subtilis*, DivIVA has been shown to migrate between the two poles of a dividing cell (Bach et al., 2014). Although MinC and MinD are not present in *B. bacteriovorus*, as they are in *B. subtilis*, DivIVA_*Bd*_ may still direct septal selection through other as-yet unidentified processes, resulting in a similar function.

Although preliminary, a number of potential interacting partners with DivIVA_*Bd*_ have been identified, and have been highlighted (Figure 6 and Supplementary Figure S5) for future investigation. Probing a *B. bacteriovorus* Bacterial Two Hybrid library identified four putative DivIVA_*Bd*_-interacting proteins beyond those encoded by the *divIVA*_*Bd*_ operon (see discussion below). Although diverse, all these proteins would be expected to change in levels in a *B. bacteriovorus* filament that has been growing for a long period in a bdelloplast, consuming oxygen and nutrients. Bd0548 MenE is a cytoplasmic protein associated with synthesis of a menaquinone to allow electron transport in more anaerobic conditions, such as those generated by continued enclosed growth of *B. bacteriovorus* inside the bdelloplast (Sharma et al., 1996). A pair of proteins Bd2106/7, associated with haem biosynthesis and disulfide bond formation in other bacteria (Zapun et al., 1993; Chan et al., 2006; Heras et al., 2009), were also found to interact with DivIVA_*Bd*_. Again, changes in synthesis of electron transport components such as Fe-S proteins and cytochromes could occur to remodel electron transport as the occupied bdelloplast was depleted for oxygen when the *B. bacteriovorus* filament reaches a greater biomass. The final DivIVA_*Bd*_ interactor identified by BTH was Bd3538, a TrmJ homolog. This is a potential oxidative stress responsive protein in Gram-negative bacteria, which is intriguing as deletion of *divIVA* in Streptococci leads to an oxidative stress phenotype (Jaroensuk et al., 2016; Ni et al., 2018). Again, this could be associated with oxidative changes in the bdelloplast toward the end of filamentous growth of the *B. bacteriovorus* and could signal the need to divide and exit the dead prey cell. While the β-galactosidase assays for interaction of the library fragments were strongly positive, whole gene interactions were less substantial, prompting future work to determine the strength and extent of these protein interactions.

The pairwise protein-protein interactions with products encoded from the *divIVA*_*Bd*_ operon, shown by BTH analysis, suggest that Bd0465 binds ParA3 (from the canonical ParAB pair) and that Bd0465 binds DivIVA (Bd0464) and Bd0466. This may coordinate the functions of DivIVA_*Bd*_ and ParA3 of the canonical ParAB complex. ParA3, but neither of the other two other ParAs, gave aberrant *B. bacteriovorus* cell length distributions when fluorescently tagged, with ParA2 only affecting gliding motility reversals when deleted and ParA1 having no measureable phenotype. Therefore the Bd0465- DivIVA_*Bd*_ -ParA3 interaction could act to synchronize the actions of DivIVA_*Bd*_ with chromosome segregation, as is thought to be the case in *M. smegmatis*, albeit through an indirect interaction (Kang et al., 2008; Ginda et al., 2013, Vijay et al., 2014). It may possibly relate to *Corynebacterium glutamicum*, where DivIVA was shown by protein-protein studies to interact with ParB (Hammond et al., 2019). We also note (Figure 5) that the *bd3904* bactofilin gene of *B. bacteriovorus* is cotranscribed with *bd3905 parB*. In *Myxococcus*, ParB interaction with bactofilin scaffolds emanating from each cell pole restrains the chromosome segregation machinery near poles until needed; something similar may operate in *B. bacteriovorus* (Lin et al., 2017).

The interaction between Bd0465, a ProC homolog, and Bd0466, an YggS homolog, has not been recorded in any other bacteria. It is possible that this interaction is a part of a signaling pathway that continues from Bd0466 to both ParA3 and DivIVA_*Bd*_. YggS proteins bind pyridoxal phosphate (PLP), and a change in the level of this molecule may signal to the division machinery to coordinate growth and septation. Recent work has suggested that changes in PLP levels are associated with changes in flux through the biosynthetic pathways for amino acids (Ito et al., 2013; Prunetti et al., 2016, Ito et al., 2019). One can see the necessity for such a signal due to *B. bacteriovorus* producing progeny at a timepoint when prey cell resources have been depleted. At a critical point in the predatory life cycle, the invading *B. bacteriovorus* cell must detect when remaining nutrients from the inside of the single prey cell are insufficient to produce another progeny cell. At this time septation must occur and the *B. bacteriovorus* cell must switch from growth and biosynthesis to the less metabolically active, prey-hunting attack phase progeny. The Bd0466 interaction with Bd0465 may represent a pivotal part of this signaling pathway. This is further supported by the constitutive expression of both *bd0465* and *bd0466*, as is the case with DivIVA_*Bd*_, and the diffuse, cytoplasmic localization of the Bd0465/0466 proteins in the predator cells. This may lead to a specific association between Bd0466 and Bd0465 occurring when PLP levels change; then in turn this could affect the Bd0465 association with ParA3, possibly releasing ParA3 for partitioning which may lead to Bd0465 association with DivIVA_*Bd*_. Demonstrating the validity of this idea and the dynamics of such a catch and release type of mechanism and its effects on cell division inside the bdelloplast, is beyond this study, but we hope that our results will prime such work.

## Conclusion

In summary we report that Gram-negative *B. bacteriovorus* HD100 uses DivIVA to define cell proportions during synchronous division from a long filamentous cell inside a dead prey bacterium. This process may involve an interaction with a canonical ParA protein and proteins that bind indicators of the nutritional contents and oxidative status of the dead prey cell.

## Data Availability Statement

All datasets generated for this study are included in the article/Supplementary Material or can be obtained by contacting the corresponding author.

## Author Contributions

DM carried out the DivIVA mutagenesis, phenotyping, some phylogenetic analyses, ParA protein tagging, gene deletion, and phenotyping work jointly with ES. LR carried out BTH library construction and analysis, further ParA3 protein tagging and phylogenetic analyses. RT carried out gene cloning for BTH analyses. CL carried out transcriptional analyses and contributed to luminsecent prey assays. AF carried out some fluorescent tagging, transcriptional analysis of bactofilin gene, and initial phylogenetic analyses. RS, DM, and AF devised the initial projects and supervised and helped to interpret the work with inputs from the other authors, drafted the manuscript with LR and edited it on receipt of comments from our co-authors.

## Conflict of Interest

The authors declare that the research was conducted in the absence of any commercial or financial relationships that could be construed as a potential conflict of interest.

## Footnotes

1 http://tree.bio.ed.ac.uk/software/figtree/
