# Supplementary material for: DivIVA Controls Progeny Morphology and Diverse ParA Proteins Regulate Cell Division or Gliding Motility in Bdellovibrio bacteriovorus
Source: Front Microbiol. 2020 Apr 21;11:542. doi: 10.3389/fmicb.2020.00542 (PMC7186360; doi:10.3389/fmicb.2020.00542)
Supplement: Supplementary file 1 [file Data_Sheet_1.pdf]

Supplementary information 1- Bioinformatic analysis of DivIVA$_{Bd}$

Features critical to the function of DivIVA in *Bacillus subtilis* were shown when the crystal structure of the N terminus was resolved (Oliva *et al.*, 2010). Of particular note were residues S16, F17, R18, G19 and Y20, which are located between two alpha helices, forming a crossed-loop that exposes them to the substrate. The N terminus is where dimerisation occurs. The crossed-loop residues were determined to be essential for membrane association, as the structure of DivIVA$_{Bsub}$ senses the negative curvature in that bacterium. Null phenotypes were observed in *B. subtilis* upon mutation of F17, R18 and G19 (Oliva *et al.*, 2010). In DivIVA$_{Bd}$ four of these residues are different from those in *B. subtilis*, although the alpha helices either side are conserved and predicted to keep the same structure (Figure 1). The sequence in DivIVA$_{Bd}$ is KMMGL, which will likely change the structure of this crossed-loop due to a loss of bonding between the dimerised proteins. The study publishing the crystal structure of DivIVA$_{Bsub}$ also showed that none of these residues are essential for dimerisation (Oliva *et al.*, 2010). Therefore, the conservation of the alpha helices suggests that DivIVA$_{Bd}$ can still dimerise.

BLAST analysis of the N terminal domain of DivIVA$_{Bd}$ showed that many DivIVA homologues have substitutes for S16 and F17. In Firmicutes this is commonly KM, although not exclusively. Indeed, the closest similarity to the N terminal domain (residues 1-36) of DivIVA$_{Bd}$ was found in members of *Bacillus* and *Lactobacillus* (up to 63% identity). In *Bacillus* F17A mutants showed diffuse cytoplasmic localisation, suggesting this residue is necessary for negative curvature sensing (Oliva *et al.*, 2010).

In DivIVA homologues, R18 is very rarely substituted with methionine, as it is in *B. bacteriovorus* (Figure 1). Even the Firmicute DivIVA homologues with the highest sequence identity to the DivIVA$_{Bd}$ N terminal domain all have a conserved R18. This residue is clearly exposed to the surrounding solvent, and the R18A mutant in *Bacillus* localised to spots in the cytoplasm (Oliva *et al.*, 2010). That *B. bacteriovorus* lacks these residues, both substituted with methionine, suggests it cannot inherently sense negative curvature. Therefore polar localisation of DivIVA$_{Bd}$ is likely to require further protein interactions.

Apart from in the "*Bdellovibrio* and like organisms" (BALOs), two other exceptions without R18 were noted, the first published for the cyanobacterial *Synechococcus elongatus* DivIVA-like protein Cdv3 (Figure 1) (MacCready *et al.*, 2017). In this context, Cdv3 does not have the first conserved alpha helix, and each of the five crossed-loop residues had been substituted, the crossed-loop sequence becoming ENDLL (Supplementary Figure 1). As with *B. bacteriovorus*, negative curvature sensing may not occur due to the loss of residues F17 and R18. Despite this, Cdv3 was observed at the midcell, recruiting MinC, suggesting another method of localisation. This could be via protein interaction. ZipN has previously been found to bind Cdv3 and many Fts proteins, and is a candidate for recruiting them to the divisome (Marbouty *et al.*, 2009), though *B. bacteriovorus* does not contain a ZipN homologue. The second exception is the Deltaproteobacterium *Geobacter*. All *Geobacter* DivIVA crossed-loops have a similar sequence (Supplementary Figure 1). An alignment shows that both alpha helices are conserved and K16 is highly conserved, but the RGY motif from DivIVA$_{Bsub}$ has been substituted with (I/L)GG(I/L). As with DivIVA$_{Bd}$ this suggests a loss of function for negative curvature sensing, but conservation of amino acids required for dimerisation.

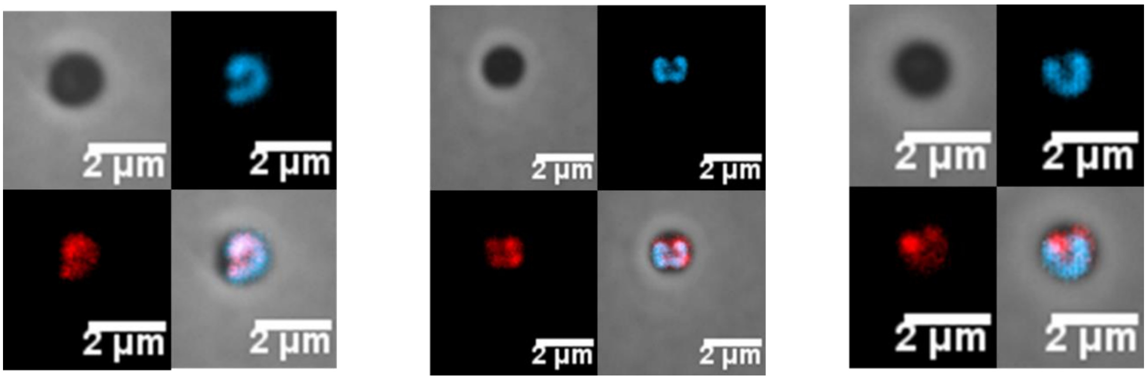

Supplementary Figure 1- Phase and epifluorescence microscopy displaying the location of DivIVA tagged with mCherry (red) during growth within *E. coli* prey cells at 3 hours. Several examples at this timepoint are presented to illustrate a diversity of growing or dividing *Bdellovibrio* filaments, with some foci and some diffuse mCherry fluorescence. The *Bdellovibrio* cytoplasm is constitutively fluorescent with Bd0064-mCerulean (blue) to visualise the cell within the bdelloplast.

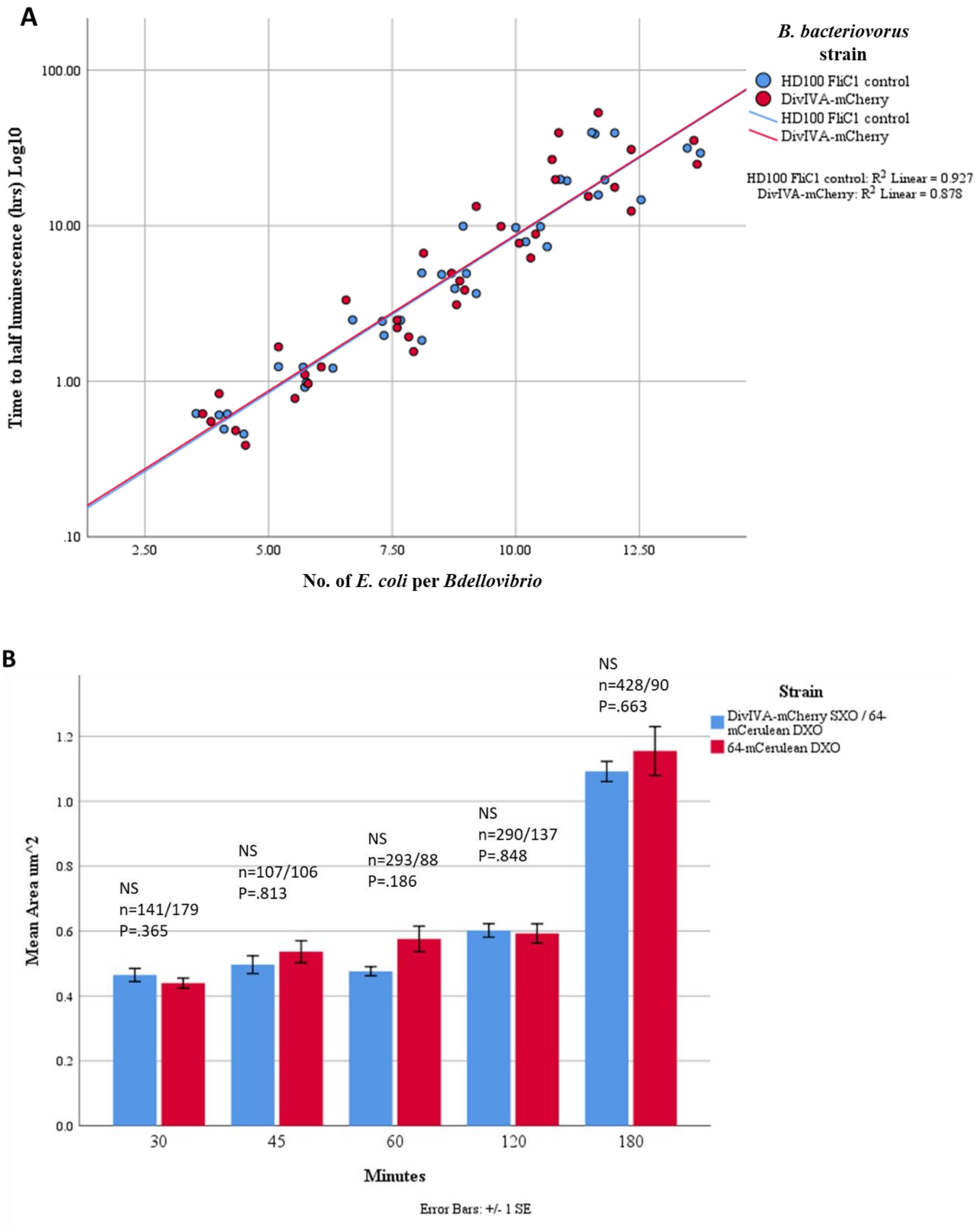

Supplementary Figure 2 (A) Luminescent prey predation assay demonstrating no difference in growth rate between DivIVA-mCherry and the control FliC1 merodiploid strain. Mann Whitney test p=0.995; n=35. (**B**) Area of intraperiplasmic *Bdellovibrio* cells measured by the MicrobeJ plugin in the cerulean fluorescence channel of DivIVA-mCherry Bd0064-mCerulean and control Bd0064-mCerulean strains show no difference in growth between the strains. *P* values by Mann-Whitney test and n values are displayed. NS- not significant.

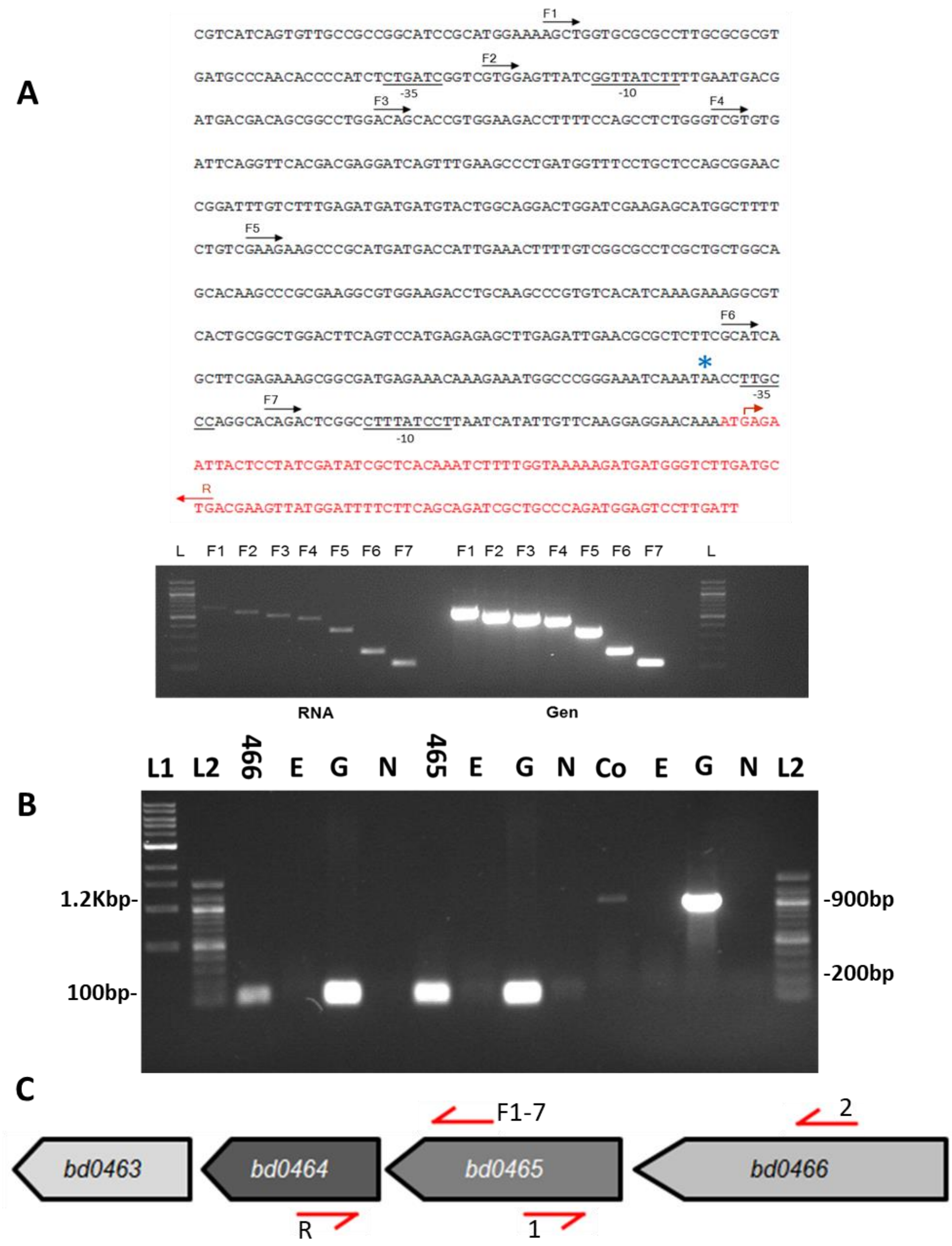

Supplementary Figure 3. RT-PCR gels showing co-transcription of *divIVA*$_{Bd}$ and neighbouring genes *bd0465* and *bd0466*. (**A**) Promoter walking to find the start of transcription for *divIVA* (*bd0464*) which shows that co-transcription occurs from *bd0465* to *divIVA*$_{Bd}$. On the sequence, Fn represents the primer used, the black sequence is upstream of *divIVA*$_{Bd}$ (including much of *bd0465*), the blue asterisk represents the stop codon for *bd0465* and the red sequence denotes *divIVA*. On the gel image, RNA represents RT-PCR assays using wild type HD100 attack phase RNA, and Gen is the positive control using genomic DNA. All primers produced products, suggesting co-transcription. (**B**) RT-PCR for co-transcription from *bd0466* to *bd0465*. L1= 1kbp ladder, L2= 100bp ladder, 466= *bd0466* primers with 3h RNA, E= negative *E. coli* control, G= positive genomic control, N= no RNA negative control, 465= *bd0465* primers with 3h RNA, Co= forward *bd0466* primer (1) and reverse *bd0465* primer (2) with 3h RNA. **C-** the positions of the primer binding sites in the operon.

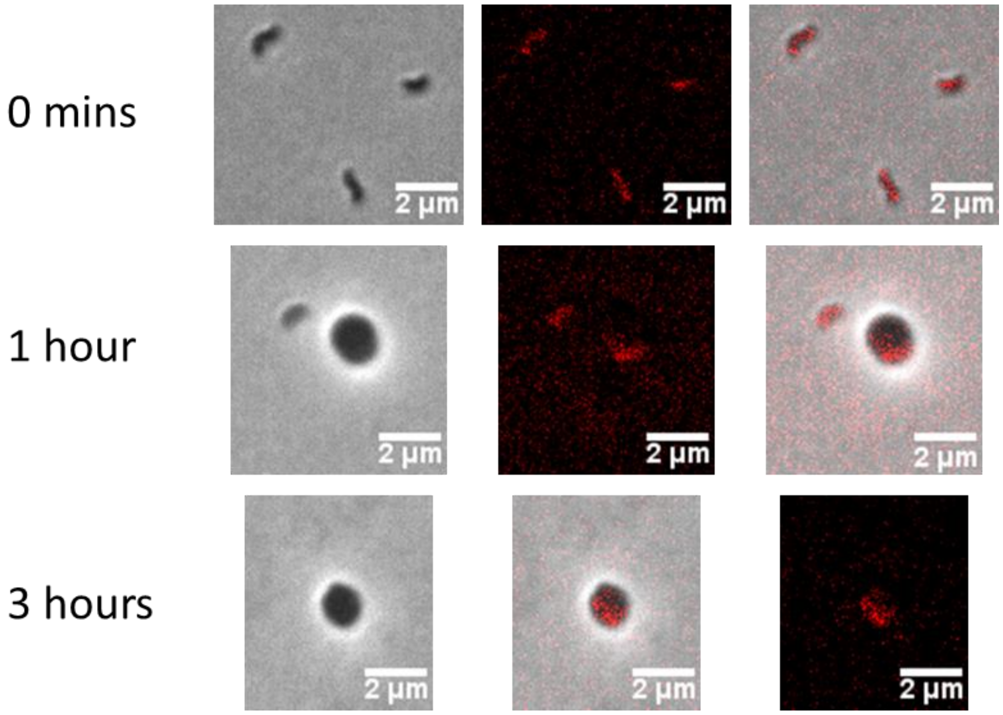

Supplementary Figure 4. Fluorescence time course of *B. bacteriovorus* expressing Bd0465-mCherry. Representative images taken at the time intervals shown after prey and *Bdellovibrio* were mixed. Bd0465-mCherry fluorescence is faint, hence high background fluorescence, but can be seen in both attack phase cells and bdelloplasts.

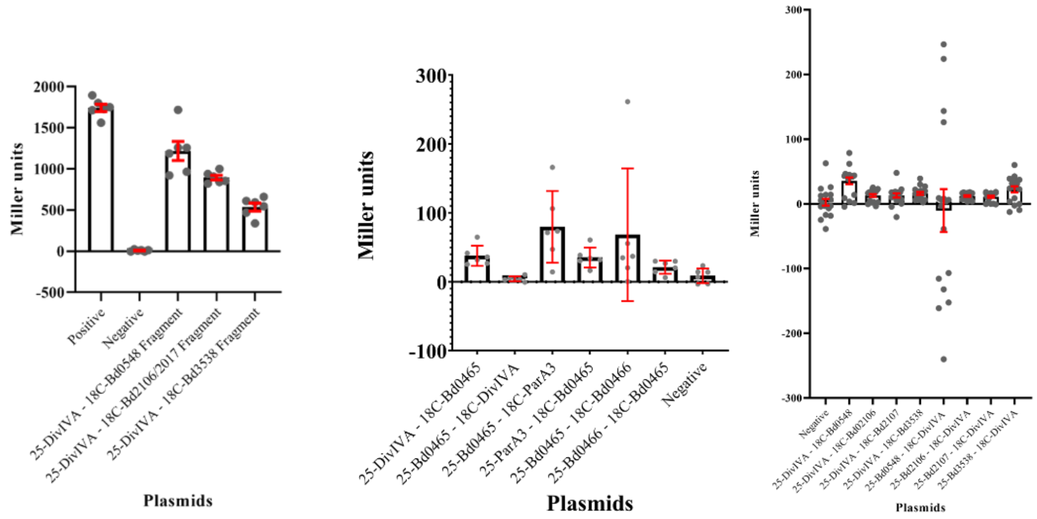

Supplementary Figure 5. Figure 6 presented with all datapoints. β-galactosidase assay results for pairwise and library screening bacterial two hybrid interactions. Points represent average Miller Units, errors bar being +/- 1 standard error, of cotransformants (n=16, two biological repeats). (**A**) shows significant interactions between $DivIVA_{Bd}$ (Bd0464) and B0465, Bd0465 and ParA3 (Bd3906), and Bd0465 and Bd0466 (all had $P<0.001$) when compared to the negative control. (**B**) shows significant interactions between protein fragments encoded from BTH library plasmids, specifically partial proteins of Bd0548, Bd2106, Bd2107 and Bd3538, with $DivIVA_{Bd}$ plasmids (all $P<0.001$). (**C**) shows results for interactions between $DivIVA_{Bd}$ and full-length proteins Bd0548, Bd2106, Bd2107 and Bd3538.

Supplementary information 2- DivIVA$_{Bd}$ BTH library screening identified four potential interacting proteins that were not Par or operonally encoded neighbour proteins

Library screens using bait plasmid pKT25-*divIVA$_{Bd}$* resulted in the identification of four library plasmids encoding potentially interacting protein fragments. Sequencing showed these to be internal gene fragments of *bd0548* (encoding 328 of 358 total residues), a fragment running across *bd2106* and *bd2107* (encoding the first 100 residues of Bd2106 and the first 133 residues of Bd2107), and *bd3538* (encoding residues 37 to 141 of 261 total).

Bd0548 is annotated as MenE, an enzyme in the Men pathway for menaquinone synthesis (Sharma *et al.*, 1996), which is a molecule involved in the respiratory electron transport chain of Gram positive bacteria, and low oxygen respiration of Gram-negative bacteria. Bd2106 is classed as a Cofac_haem_bdg domain containing protein, thought to be involved in haem uptake. Bd2107 is a DsbA homologue, required for the formation of disulfide bonds in some proteins as they enter the periplasm (Heras *et al.*, 2009, Zapun *et al.*, 1993).

Bd3538 is annotated as a TrmH but it shows greater homology to TrmJ, which is a cytoplasmic tRNA (cytidine/uridine-2'-O-)-methyltransferase. TrmJ catalyses the formation of 2'O-methylated cytidine (Cm32) or 2'O-methylated uridine (Um32) at position 32 in tRNA. Deletion of this gene in *Pseudomonas aeruginosa* increases susceptibility to oxidative stress from hydrogen peroxide (Jaroensuk *et al.*, 2016). Interestingly, in *Streptococcus suis*, deletion of *divIVA* leads to the same phenotype (Ni *et al.*, 2018).

Full-length copies of these four genes were tested in a pairwise fashion with the *divIVA$_{Bd}$* BTH plasmids. Potential interactions were measured with the one step β-galactosidase assay, shown in Figure 6. Compared to the negative control, all three library fragment products showed a significant interaction with a large effect (Miller units>500, P<0.001). The full-length products, however, showed significant interactions with a small effect size (Miller units for significant interactions range between 13 and 37) (Figure 6).

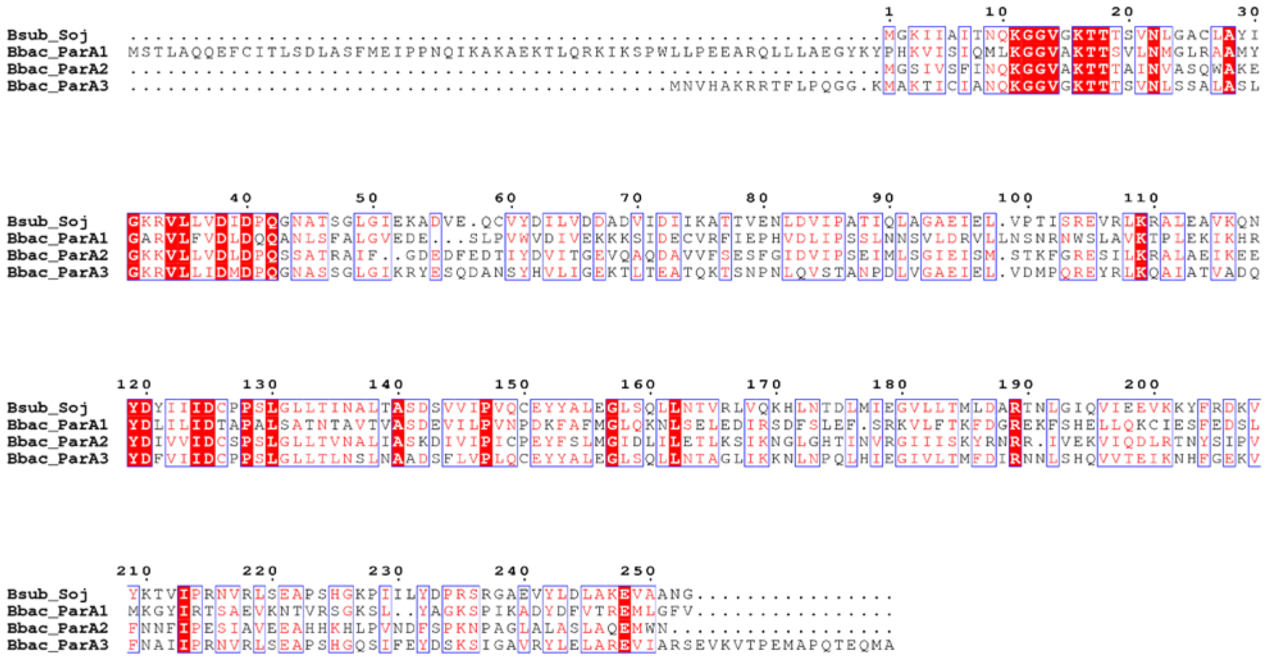

1:	Bsub_Soj	100.00	27.94	35.48	57.71
2:	Bbac_ParA1	27.94	100.00	24.28	22.73
3:	Bbac_ParA2	35.48	24.28	100.00	32.93
4:	Bbac_ParA3	57.71	22.73	32.93	100.00

Supplementary Figure 6. An alignment of *Bacillus subtilis* Soj (ParA) and *B. bacteriovorus* ParA homologues Bd1326 (ParA1), Bd2331 (ParA2) and Bd3906 (ParA3). In the alignment (**A**) residues are boxed and highlighted red for identity or coloured red for similarity. (**B**) shows an identity matrix produced by Clustal2.1 denoting the number of identical residues, as a percentage, between each of the aligned sequences.

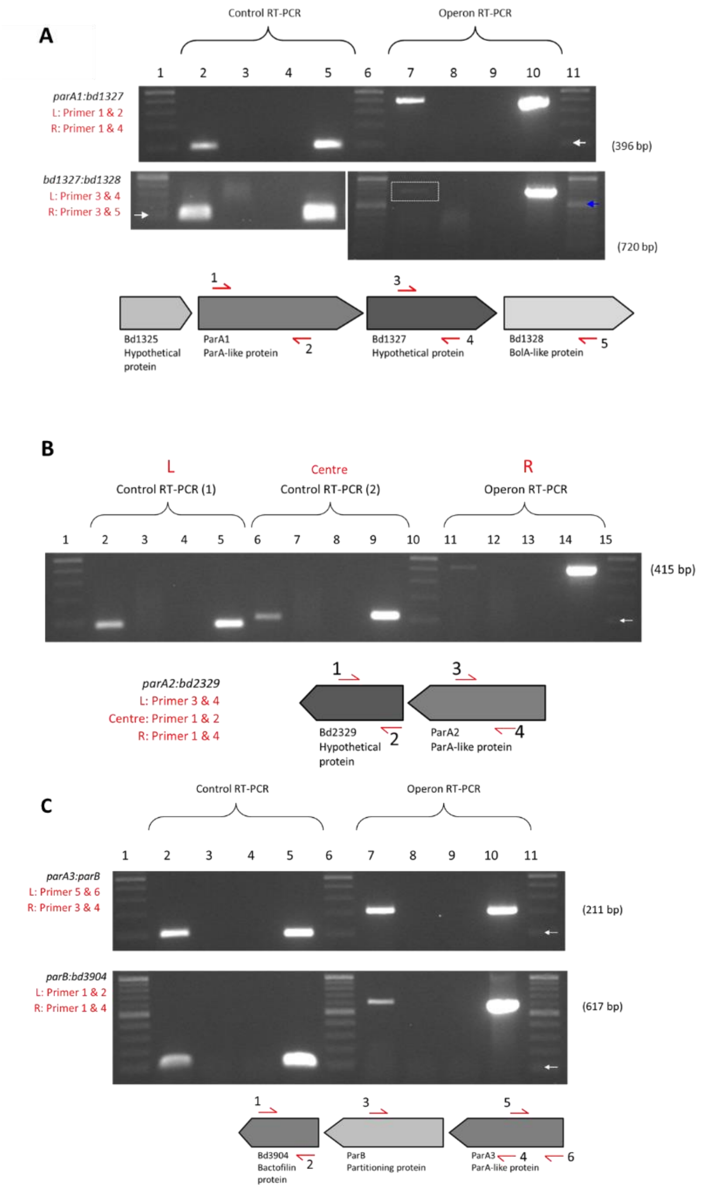

Supplementary Figure 7. RT-PCR gels showing transcription across ParA homologues and their neighbouring genes. (A) shows transcription from *parA1* across *bd1327*, and from *bd1327* to *bd1328*, with an area overlapping between the two sets of primers. (B) shows transcription across *parA2* and *bd2329*. (C) shows transcription from *parA3* across *parB* and *bd3904*.

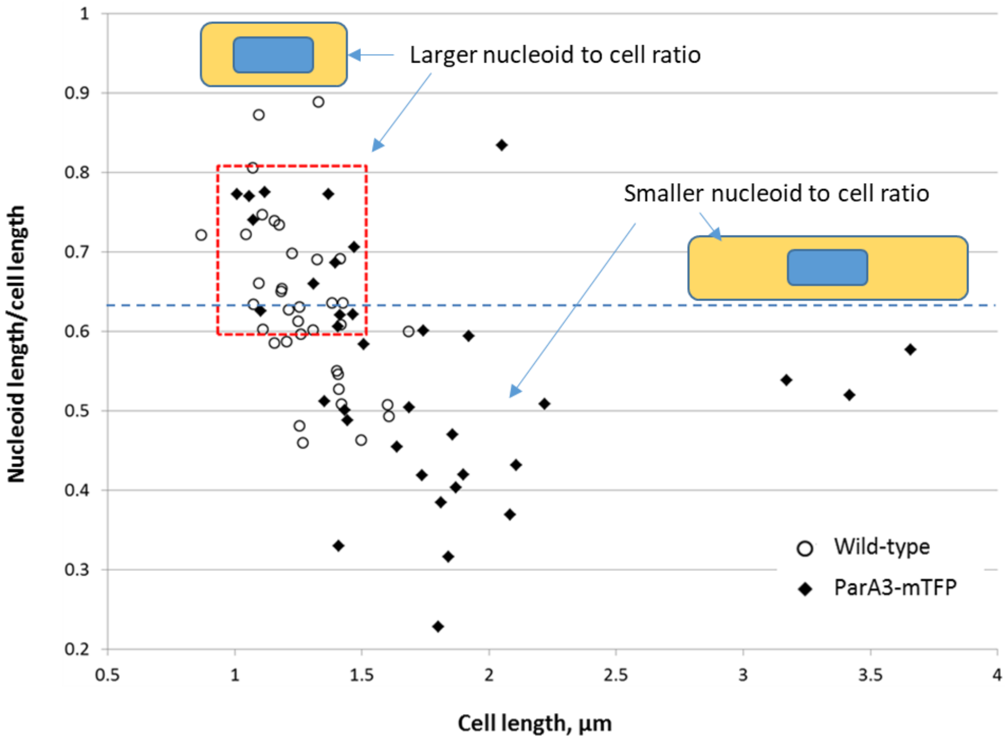

Supplementary Figure 8. Nucleoid size plotted as a function of the cell length of attack phase cells expressing ParA3-mTFP. Nucleoid/cell length ratio plotted against cell length (µm) for kanamycin-resistant wild-type control (*fliC1* merodiploid, open circles) and ParA3-mTFP cells (closed diamonds). Red box shows the typical clustering of wild-type cells as previously described (Butan, Hartnell et al. 2011). The ratio is significantly lower in cells with ParA3-mTFP (n=35, P<0.05). Data are from three biological repeats.

Supplementary Information 3- ParA2 has a role in gliding motility

Deletion strains *ΔparA1* and *ΔparA2* showed no difference in growth rate or morphology as HI or HD cells. Both the strains retained flagellar motility in liquid culture, and gliding motility upon a solid surface (1% agarose/CaHEPES). However, gliding motility in the *ΔparA2* strain, but not in the *ΔparA1* strain, was markedly different phenotypically to wild-type *Bdellovibrio* cells. After an initial period of backward-forward movement in the first hour of gliding motility, movement of the wild-type HD100 cells altered to predominantly unidirectional movement in the second hour. In contrast, in the second hour of gliding, *ΔparA2* continued to make a higher number of reversals (Supplementary Figure 9). ParA1-mCherry showed weak fluorescence and varied location in attack-phase *Bdellovibrio* cells, and the majority of cells (71.1%, n=647) did not display any visible fluorescence (Supplementary Figure 10). ParA2-mCherry fluorescence was much more abundant, with the majority of cells (83.74%, n=824) showing cytoplasmic fluorescence. Some ParA2-mCherry cells (14.20%) displayed bi-lobed foci, where a cytoplasmic focus appeared to be bisected. The percentage of cells with bipolar foci increased after initial incubation on an agarose surface, from 0.77% of cells to 10.94% (n=521). Fluorescent time-lapse images revealed that foci at both poles correlated strongly with non-gliding cells (90.7% of cells did not glide) while cells with non-bipolar fluorescence showed strong correlation with gliding cells (61.9% of cells displayed gliding) (Supplementary Figure 11).

A

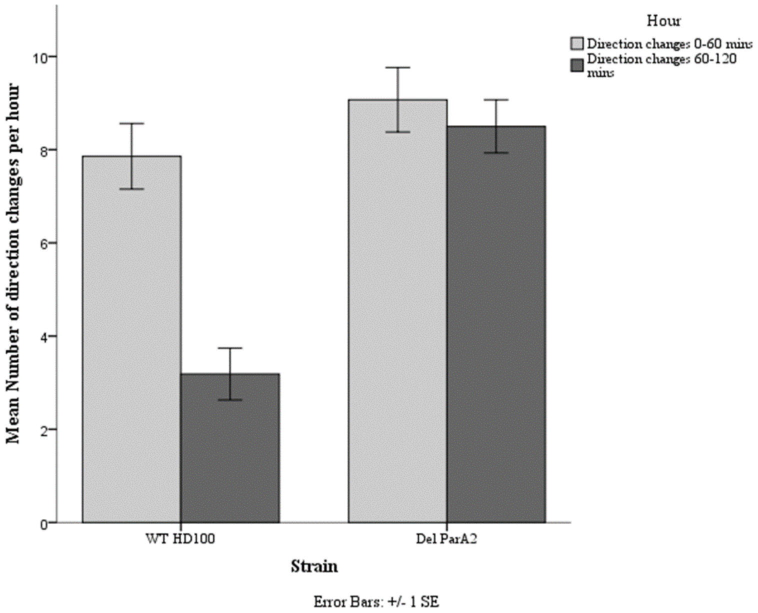

B

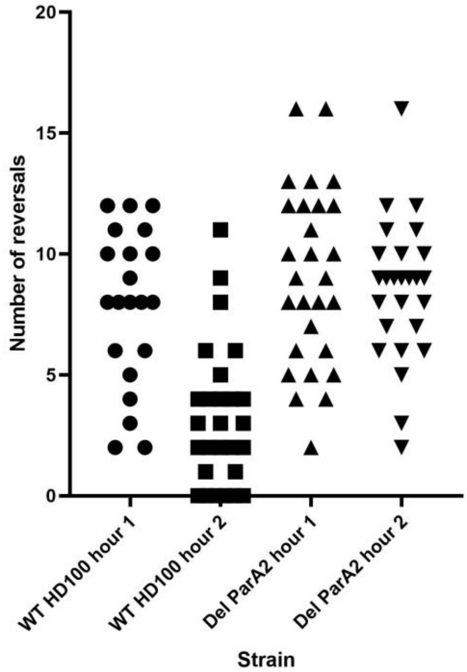

Supplementary Figure 9- Mean number of reversals of gliding cells of wild type *B. bacteriovorus* HD100 and *parA2* deletion strain. The initial period of frequent direction reversal movement in the first hour of gliding motility is followed by sustained unidirectional movement in the second hour in wild type, but not in the Δ*parA2* deletion strain (n=26, P<0.001). This is shown as mean values in (A) with error bars representing +/- 1 SEM. Individual data points are shown in (B). Data are from three biological repeats.

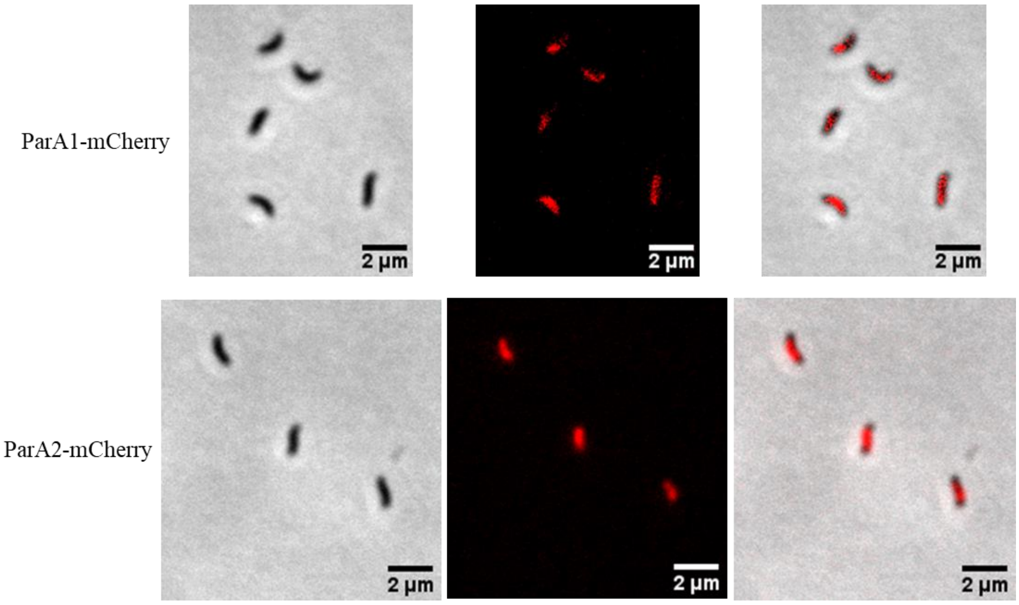

Supplementary Figure 10. Attack phase cells expressing ParA1-mCherry and ParA2-mCherry. Representative images of attack phase cells expressing ParA1-mCherry appear to have cytoplasmic fluorescent localisation, whereas ParA2-mCherry fluorescence is central within the cell.

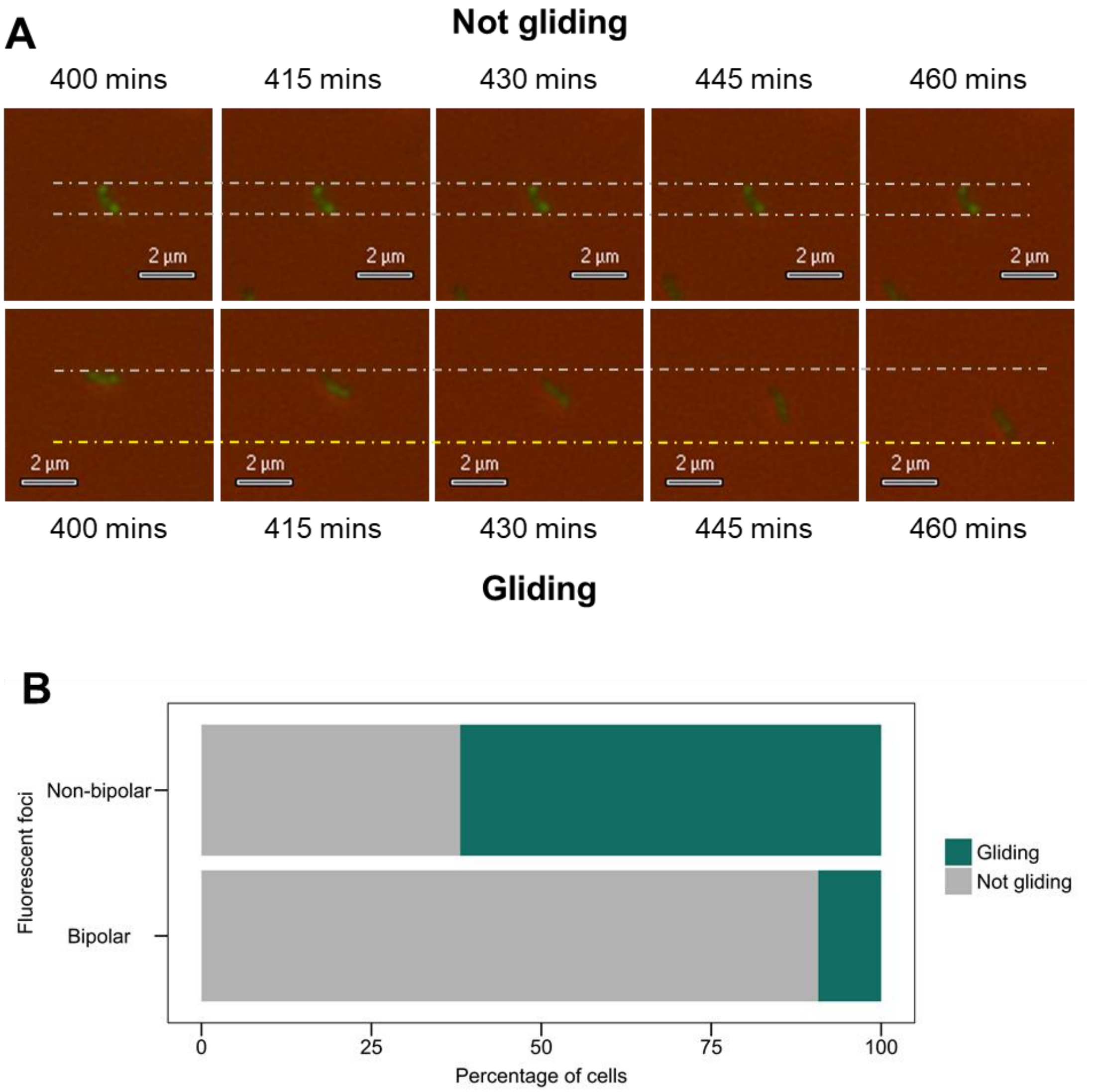

Supplementary Figure 11. Gliding motility of fluorescent and non-fluorescent ParA2-mCherry expressing cells. (A) Cell with bipolar fluorescence, not gliding (top); cell with non-bipolar fluorescence that is gliding (bottom). (**B**) 90.7% of bipolar cells did not glide (grey), whilst 61.9% (green) of non-bipolar cells were gliding (n=561, P<0.0001, Fisher's Exact Test). Data are from three biological repeats.

Supplementary Information 4

Using the top homologous sequences from pBLAST, protein trees were constructed to show the relationship between the three ParA homologues in *B. bacteriovorus* and other prokaryotes (Supplementary Figure 12). As expected, canonical ParA3 is most homologous to other BALOs and Deltaproteobacteria, such as *Geobacter* and *Desulfuromonas*. Beyond this group, however, it is closest to a group of Firmicutes, specifically *Listeria* and *Paenibacillus*. Interestingly pBLAST analysis of $DivIVA_{Bd}$ gives similar results, with a group of Deltaproteobacteria being closest, followed by several *Paenibacillus* (*Paenibacillus* sp. UNC496MF DivIVA homologue had 36% identity and 60% similarity). ParA1, which bears the least similarity to ParA3, shows greatest homology to Gammaproteobacteria, namely *Piscerickettsia* and *Legionella*. It must be noted, however, that bootstrapping values for much of this tree are low. ParA2 returned very few homologous sequences. The only three that were non-BALOs were Planctomycetes and Omnitrophica, members of the Planctomycetes, Verrucomicrobia, and Chlamydiae (PVC) group.

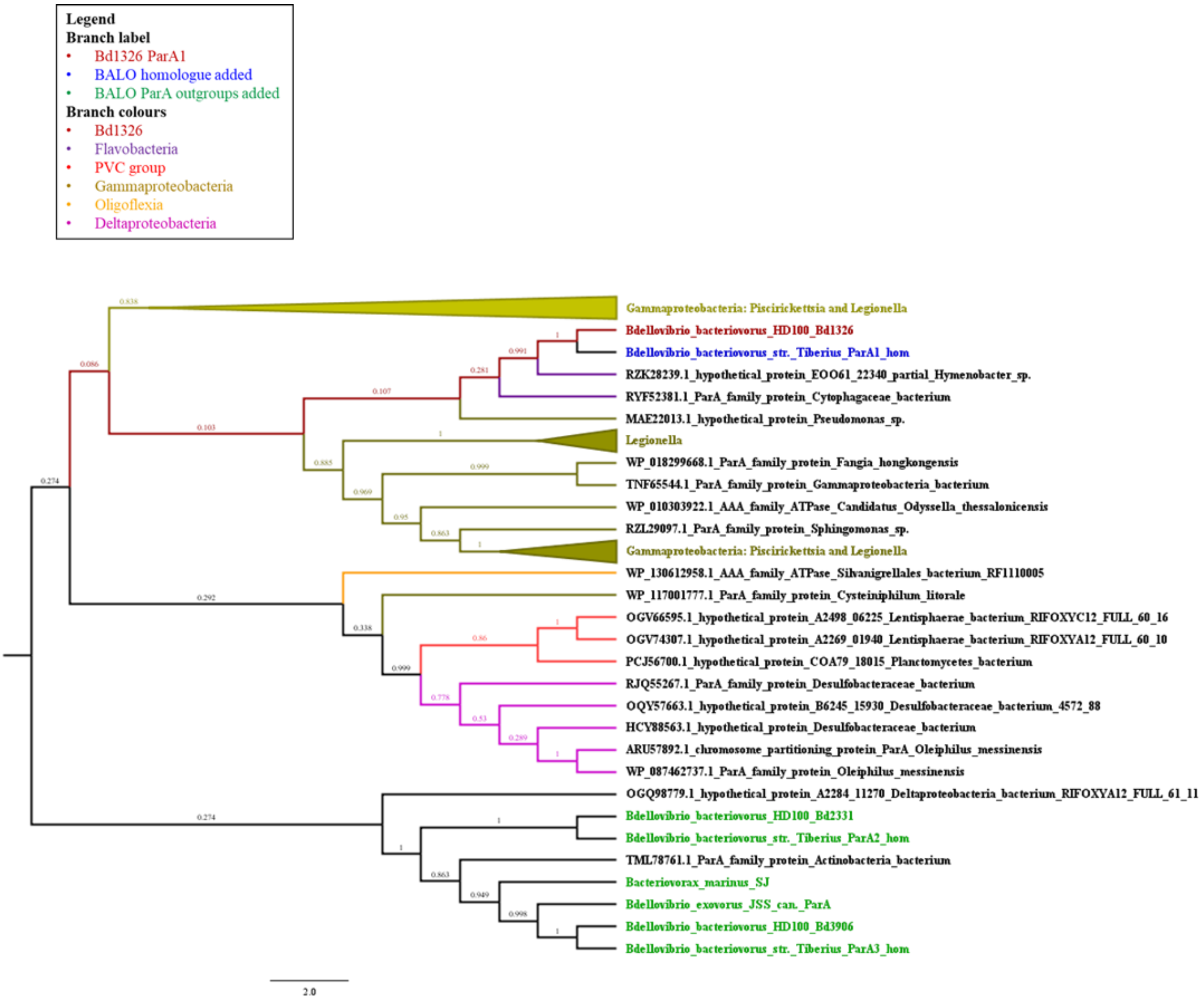

Supplementary Figure 12A. Phylogenetic trees of each ParA homologue in *B. bacteriovorus*. Protein trees for Bd1326 ParA1 (**A**), Bd2331 ParA2 (**B**) and ParA3 Bd3906 (**C**) are shown. Trees were made using sequences from pBLAST using standard settings. Sequences were aligned and trees produced in MEGA software, using the Maximum Likelihood method, bootstrapped 1000 times (indicated as the branch values). Trees were visualised in Figtree software.

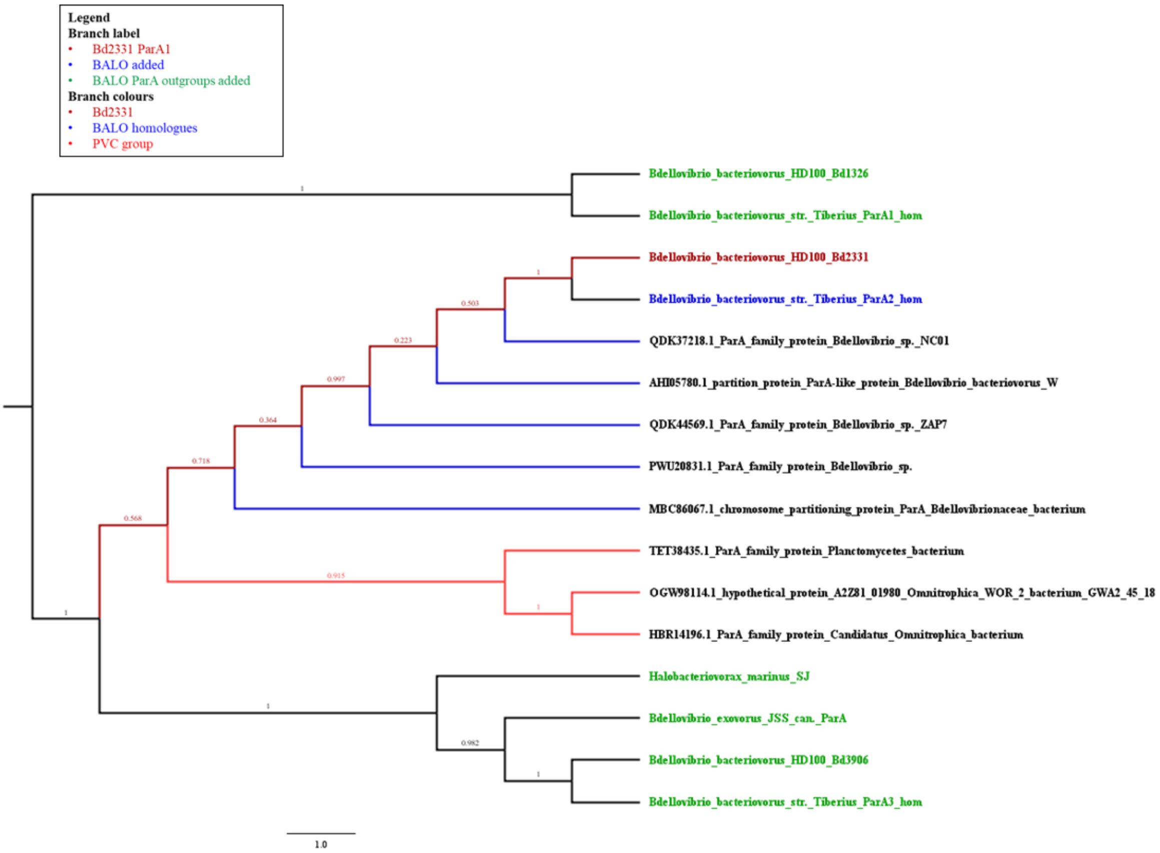

Supplementary Figure 12B. Phylogenetic trees of ParA2.

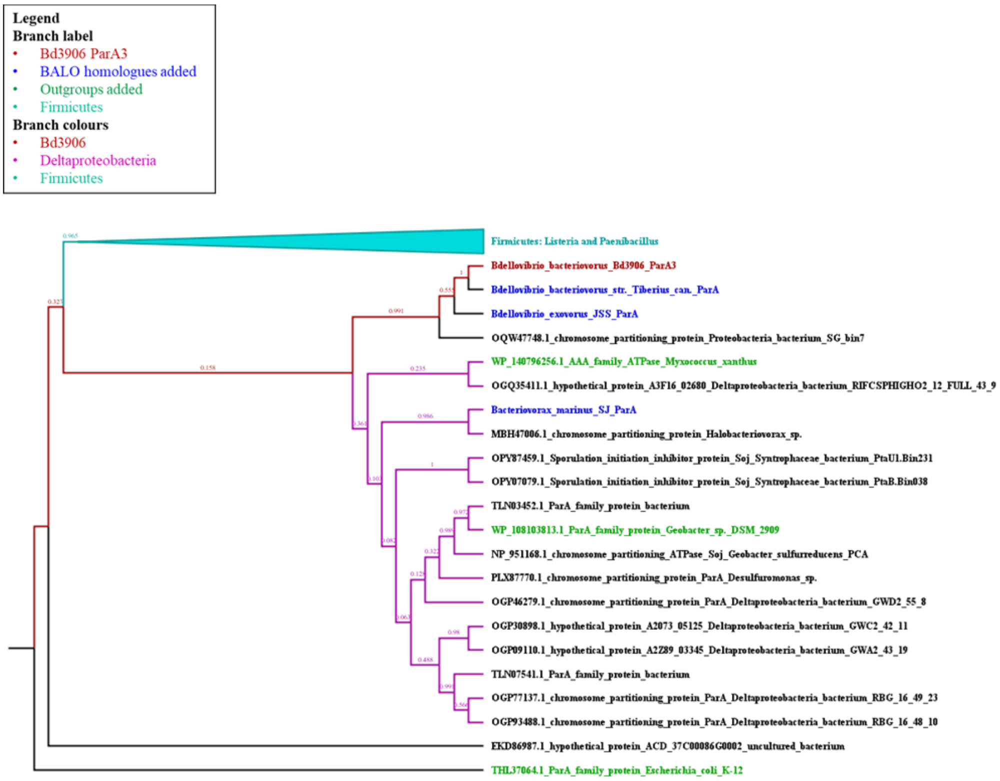

Supplementary Figure 12B. Phylogenetic trees of ParA3.

Bdellovibrio bacteriovorus		
HD100	Wild-type *Bdellovibrio* strain, genome sequenced	(Rendulic, Jagtap et al. 2004)
HID13	Host-independent derivative of HD100	(Lambert, Ivanov et al. 2010)
HID26	Host-independent derivative of HD100	(Lambert, Ivanov et al. 2010)
HD100 DivIVA-mCherry	HD100 with pK18::DivIVA-mCherry integrated at *bd0464* (*divIVA*) locus	This study
HD100 DivIVA-mCherry HI	Host-independent derivative of HD100 DivIVA-mCherry	This study
HD100 Δ*divIVA*	HD100 with *bd0464* markerless deletion	This study
HD100 pSUP404.2	HD100 with empty pSUP404.2 vector	This study
ΔdivIVA pSUP404.2	HD100 Δ*divIVA* with empty pSUP404.2 vector	This study
ΔdivIVA pSUP404.2 DivIVA	HD100 Δ*divIVA* with pSUP404.2 DivIVA complementation vector	This study
ΔdivIVA pSUP404.2 DivIVA(A78T)	HD100 Δ*divIVA* with pSUP404.2 DivIVA (A78T) complementation vector	This study
HD100 pSUP404.2 DivIVA(A78T)	HD100 with pSUP404.2 DivIVA (A78T) complementation vector	This study

Table 1A. Strains used in this study.

HD100 Bd0465-mCherry	HD100 with pK18 *bd0465-mCherry* integrated at *bd0465* locus	This study
Hd100 Bd0466-mTFP	HD100 with pK18 *bd0466-mTFP* integrated at *bd0466* locus	This study
HD100 ParA1-mCherry	HD100 with pK18 *parA1-mCherry* integrated at *bd1326* (parA1) locus	This study
HD100 ParA2-mCherry	HD100 with pK18 *parA2-mCherry* integrated at *bd2331* (*parA2*) locus	This study
HD100 ParA3-mTFP	HD100 with pK18 *parA3-mTFP* integrated at *bd3906* (*parA3*) locus	This study
HD100 Δ*parA1*	HD100 with *bd1326* markerless deletion	This study
HD100 Δ*parA2*	HD100 with *bd2331* markerless deletion	This study
HD100 *fliC1*/*fliC1*::Kn^r	Merodiploid HD100 with both wild-type and kanamycin cassette-interrupted copy of *fliC1* (*bd0604*)	(Morehouse, Hobley et al. 2011)

Table 1B Strains used in this study.

Escherichia coli		
DH5α	F^- *endA1 hsdR17* ($r_k^-mk^-$) *supE44* thi-1 *recA1 gyrA* (Nal^r) *relA1* Δ(*lacIZYA-argF*) U169 *deoR* (*80dlacΔ*(*lacZ*)M15); used as a cloning strain	(Hanahan 1983)
S17-1	*thi,pro,hsdR*$^-$,*hsdM*$^+$,*recA*; integrated plasmid RP4-Tc::Mu-Kn::Tn7; used as donor for conjugating plasmids into *B. bacteriovorus*	(Simon, Priefer et al. 1983)
S17-1::pMAL_p2-mCherry	S17-1 with Amp^r plasmid containing *mCherry* gene with a *malE* signal sequence for localised periplasmic fluorescence	(Fenton, Kanna et al. 2010)
S17-1 pCL100	S17-1 with Kn^r plasmid encoding *luxCDABE* for luminescence for predation assays of Kn^r *B. bacteriovorus* strains	(Lambert, Smith et al. 2003)
S17-1 lux	S17-1 strain expressing the strawberry lux gene at the attTn7 locus.	Gift from Dr. Phil Hill.
BTH101	F^-,Δ*cya99*, *galE15*, *galK16*, *rpsL1*, *hsdR2*, *mcrA1*, *mcrB1*	(Karimova, Dautin et al. 2008)

Table 1C Strains used in this study.

RT-PCR primer	Primer sequence
divIVA_RT_F	GGAATACAAAGAGCGCGATC
divIVA_RT_R	TCAATTTCGCTTCACGGTCG
465_RT_F	TCACGACGAGGATCAGTTTG
465_RT_R	CCAGTCCTGCCAGTACATCA
466_RT_F	GATTGGCCACCTTCAAAAGA
466_RT_R	CCTGAGCCAGTTCCAGAGAG
parA1_RT_F	GCAAAAGAACCTGAGCGAAC
parA1_RT_R	TGAACTTCTCACGTCCATCG
parA2_RT_F	AGAACGGTCTTGGTCACACC
parA2_RT_R	TCCTGGATGACCTTTTCCAC
parA3_RT_F	GAAAGCCAGGATGCGAATAG
parA3_RT_R	TAGCCGTGGATACTTGAAGG
bd3904_RTPCR_F	CGGGTTGAGGGAAATCTCTT
bd3904_RTPCR_R	CCTTCGTCGATACGCAGACT
parA_operon_F	GATAGCACGCTCTGAAGTGA
parB_operon_R	TAATAGATGGGGCAGCAGCA
dnaKF	TGAGGACGAGATCAAACGTG
dnaKR	AAACCAGGTTGTCGAGGTTG

Table 2A Primers used in this study.

Promoter walking primers	Primer sequence
DivProm_F1	AGCTGGAGCGTTACGTTCAA
DivProm_F2	GTGGAGTTATCGGTTATCTT
DivProm_F3	ACAGCACCGTGGAAGACCTT
DivProm_F4	TCGTGTGATTCAGGTTCACG
DivProm_F5	GAAGAAGCCCGCATGATGAC
DivProm_F6	GCATCAGCTTCGAGAAAGCG
DivProm_F7	CAGACTCGGCCTTTATCCTT
DivProm_R	CAGCATCAAGACCCATCATC

Table 2B Primers used in this study.

Fluorescent tag primers	Primer sequence
divIVA_tag_F	GGAATTCCACAGACTCGGCCTTTATC
divIVA_tag_R	GGGGTACCTTCAGCAGAAAGAGGGGACAC
465_tag_F	GTACTGGAATTCATGAATCCCTTGTTGAAATC
465_tag_R	AGTCAGGGTACCTTTGATTTCCCGGGCCATTT
466_tag_F	GTACTGGAATTCATGGCATTAAAAGAGATCAC
466_tag_R	AGTCAGGGTACCCCCTTTGGCGGGACGTTCGC
bd1326_tag_F	GGAATTCTATTTGTGGACTTGGATCAGC
bd1326_tag_R	GGGGTACCAACAAATCCCAACATTTCACG
bd2331_tag_F	GGAATTCTCGAAGACACAATCTATGACG
bd2331_tag_R	GGGGTACCATTCCACATTTCCTGAGCAAG
Bd2329_tag_F	CGTCGCGAATTCGCACAACCCCTTGTTTCTAG
bd3906_tag_F	GGAATTCATGCGAATAGCTACCATGTGC
bd3906_tag_R	GGGGTACCTGCCATTTGTTCTGTCTGTG

Table 2C Primers used in this study.

Deletion and complementation primers	Primer sequence
divIVA_KO_F1	CGGGGTACCTGAATGAGCTGTCAATGGGC
divIVA_KO_R2	GCTCTAGACAACCCTGCTTCGGATACCG
divIVA_KO_F2	GGAACAAAATGAGAGGATTCTCTGCTGAATAATCAGCACG
divIVA_KO_R1	CGTGCTGATTATTCAGCAGAGAATCCTCTCATTTTGTTCC
parA1_KO_F1	GGGGTACCAGGTTCCCGGTGTCAGTTGC
parA1_KO_R2	GCTCTAGACGCCCTAGGACCGACGATCG
parA1_KO_F2	CTGACGGAGGTCCTTCATGGGATCCTTGGGATTTGTTTAAAAGG
parA1_KO_R1	CCTTTTAAACAAATCCCAAGGATCCCATGAAGGACCTCCGTCAG
ParA2_KO_F1	TCCCCCCGGGAAACATTGAGGAATGCGATC
ParA2_KO_R2	CTGTAGCATGCTTGACGAAGACGAACACGGC
ParA2_KO_F2	CACGAAGGGAATCGTGTATGGGATCCCAGGAAATGTGGAATTGAAA
ParA2_KO_R1	TTTCAATTCCACATTTCCTGGGATCCCATACACGATTCCCTTCGTG
divIVA-pSUP404_F	GGAATTCCTGCAAGCCCGTGTCACATC
divIVA-pSUP404_R	GGAATTCTGTGAGGACCCACAACTTCG
divIVA_A78T_F	CAACTCAGATGACCGACCGCCTTCG
divIVA_A78T_R	CGAAGGCGGTCGGTCATCTGAGTTG

Table 2D Primers used in this study.

Bacterial Two Hybrid primers	Primer sequence
divIVA_BTH_F	ccagacTCTAGACATGAGAATTACTCCTATCGA
divIVA_BTH_R	aatgtgGGTACCTTATTCAGCAGAAAGAGGGG
parA_BTH_F	ccagacTCTAGACATGGCAAAAACAATCTGCAT
parA_BTH_R	aatgtgGGTACCTTATGCCATTTGTTCTGTCT
parB_BTH_F	ccagacTCTAGACATGTCTGATATTGCTGTAGA
parB_BTH_R	aatgtgGGTACCTTACTGCCATCCTTCTTTAA
Bd0466_BTH_F	ccagacTCTAGACATGGCATTAAAAGAGATCAC
Bd0466_BTH_R	aatgtgGGTACCCTACCCTTTGGCGGGACGTT
Bd0465_BTH_F	ccagacTCTAGACATGAATCCCTTGTTGAAATC
Bd0465_BTH_R	aatgtgGGTACCTTATTTGATTTCCCGGGCCA

Table 2E Primers used in this study.

References

HERAS, B., SHOULDICE, S. R., TOTSIKA, M., SCANLON, M. J., SCHEMBRI, M. A. & MARTIN, J. L. 2009. DSB proteins and bacterial pathogenicity. *Nature Reviews Microbiology,* 7**,** 215-225.

JAROENSUK, J., ATICHARTPONGKUL, S., CHIONH, Y. H., WONG, Y. H., LIEW, C. W., MCBEE, M. E., THONGDEE, N., PRESTWICH, E. G., DEMOTT, M. S., MONGKOLSUK, S., DEDON, P. C., LESCAR, J. & FUANGTHONG, M. 2016. Methylation at position 32 of tRNA catalyzed by TrmJ alters oxidative stress response in Pseudomonas aeruginosa. *Nucleic Acids Research,* 44**,** 10834-10848.

MACCREADY, J. S., SCHOSSAU, J., OSTERYOUNG, K. W. & DUCAT, D. C. 2017. Robust Min-system oscillation in the presence of internal photosynthetic membranes in cyanobacteria. *Molecular Microbiology,* 103**,** 483-503.

MARBOUTY, M., SAGUEZ, C., CASSIER-CHAUVAT, C. & CHAUVAT, F. 2009. ZipN, an FtsA-like orchestrator of divisome assembly in the model cyanobacterium Synechocystis PCC6803. *Molecular Microbiology,* 74**,** 409-420.

NI, H., FAN, W. W., LI, C. L., WU, Q. Q., HONGFEN, H. F., HUI, D., ZHENG, F., ZHU, X. H., WANG, C. J., CAO, X. R., SHAO, Z. Q. & PAN, X. Z. 2018. Streptococcus suis DivIVA Protein Is a Substrate of Ser/Thr Kinase STK and Involved in Cell Division Regulation. *Frontiers in Cellular and Infection Microbiology,* 8.

OLIVA, M. A., HALBEDEL, S., FREUND, S. M., DUTOW, P., LEONARD, T. A., VEPRINTSEV, D. B., HAMOEN, L. W. & LOWE, J. 2010. Features critical for membrane binding revealed by DivIVA crystal structure. *Embo Journal,* 29**,** 1988-2001.

SHARMA, V., HUDSPETH, M. E. S. & MEGANATHAN, R. 1996. Menaquinone (vitamin K-2) biosynthesis: Localization and characterization of the menE gene from Escherichia coli. *Gene,* 168**,** 43-48.

ZAPUN, A., BARDWELL, J. C. A. & CREIGHTON, T. E. 1993. The Reactive and Destabilizing Disulfide Bond of Dsba, a Protein Required for Protein Disulfide Bond Formation Invivo. *Biochemistry,* 32**,** 5083-5092.
